# Biophysical and biochemical studies support PHD inhibitor development as a TPI deficiency therapy

**DOI:** 10.1242/jcs.264664

**Published:** 2026-05-13

**Authors:** Presley Roberts, Joseph R. Figura, Kaitlin McClure, Grace Coleman, Cindy Cui, Riley Sawka, Laura L. Vollmer, Andreas Vogt, Ariana J. Jou, Andrew P. VanDemark, Michael J. Palladino

**Affiliations:** ^1^ Department of Pharmacology & Chemical Biology, University of Pittsburgh School of Medicine, Pittsburgh, PA 15260, USA; ^2^ Pittsburgh Institute for Neurodegenerative Diseases, University of Pittsburgh School of Medicine, Pittsburgh, PA 15260, USA; ^3^ Department of Biological Sciences, University of Pittsburgh, Pittsburgh, PA 15260, USA; ^4^ Department of Computational & Systems Biology, Organ Pathobiology and Therapeutics Institute. University of Pittsburgh School of Medicine, Pittsburgh, PA 15260, USA

**Keywords:** Triosephosphate isomerase, TPI, TPI deficiency, Hypoxia-inducible factor, HIF, Prolyl hydroxylase domain inhibitors, PHDIs, Crystallography, Glycolysis

## Abstract

Triosephosphate isomerase deficiency (TPI Df) is an ultra-rare genetic enzymopathy. Previously, the *TPI^R5G^* allele was found to cause TPI Df when combined with a null allele. Here, we report a 1.15 Å TPI^R5G^ crystal structure providing insight into disease pathogenesis. Previously, we conducted a high-throughput screen that identified TPI-inducing compounds, including predicted hypoxia inducible factor (HIF) inducers. We have investigated repurposing HIF activators/prolyl hydroxylase domain inhibitors (PHDIs) as TPI Df treatments. We tested the efficacy of these compounds in cells from individuals with TPI Df. Our results demonstrate that PHDIs increase TPI protein levels and TPI activity, suggesting they should be further developed for TPI Df. RNA-sequencing and reverse transcription quantitative polymerase chain reaction (RT-qPCR) experiments were performed to analyze PHDI-induced gene expression changes. We discovered that chronic PHDI treatment results in HIF1-antisense 2 (*HIF1-AS2*) activation, which operates as a negative feedback loop in the HIF pathway. These results demonstrate that repurposing PHDIs for TPI Df is a promising avenue of research deserving further investigation. Our results also suggest that PHDIs may also benefit dozens of other heritable disease conditions if treatment avoids *HIF1-AS2* activation.

## Teaser

**Summary:** Analysis of human fibroblasts with a *TPI^R5G^* mutation demonstrate that prolyl hydroxylase domain inhibitors, such as vadadustat, improve protein and activity levels *in vitro*, highlighting them as a possible treatment strategy for triosephosphate isomerase deficiency.

## INTRODUCTION

Triosephosphate isomerase deficiency (TPI Df) is an autosomal recessive, ultra-rare multisystem disorder caused by pathogenic mutations in the *TPI1* gene (Williams et al., 2025). Pathogenic missense alleles that cause TPI Df encode TPI protein that remains functional but has reduced cellular stability and low steady-state levels. This disease is characterized by hemolytic anemia, severe neuromuscular dysfunction and premature death. Diagnoses typically occur between ages 1 and 5 years old. In typical cases of TPI Df, such as those homozygous for the ‘common’ *TPI^E105D^* missense mutation, individuals are seemingly unaffected at birth, but miss growth milestones, are diagnosed with hemolytic anemia and have increased rates of infection. Neuronal dysfunction and progressive muscle degeneration are symptoms that appear as pathogenesis advances. In severe cases of this disease, such as in cases where the *TPI^E105D^* mutation is combined with a null allele, there is presentation of neonatal onset of disease symptoms, and lethality in infancy (Williams et al., 2025). Pathogenic TPI Df missense mutations predominantly affect the stability of the mutant TPI protein, which underlies disease pathogenesis. Currently, there are no medical treatments for this disease aside from bone marrow transplantation to correct the anemia, dietary interventions (such as the ketogenic diet) and supportive care.

TPI is an enzyme essential for glycolysis that catalyzes the interconversion of dihydroxyacetone phosphate (DHAP) and glyceraldehyde-3-phosphate (G3P). This step is crucial to the glycolysis process, as G3P can be metabolized by the ATP-yielding steps in glycolysis, making this necessary for the maximum energetic production of glycolysis and gluconeogenesis. There is significant evidence that DHAP accumulation contributes to disease pathogenesis by elevating methylglyoxal, a highly reactive, toxic α-dicarbonyl that modifies cellular macromolecules including TPI (de la Mora-de la Mora et al., 2024; Farrera and Galligan, 2022; Trujillo and Galligan, 2023).

*TPI^R5G^* was previously reported as a novel mutation that leads to TPI Df as a compound heterozygote with a frame-shift allele (*TPI^f.s.^*) and results in locomotor and neurologic impairment, with a lack of anemia (Figura et al., 2025). The *TPI^R5G^* allele encodes a protein that retains nearly wild-type levels of protein activity, but has low steady-state levels that ultimately lead to disease (Figura et al., 2025). Small molecules that work to increase mutant protein levels represent one strategy to develop treatments for TPI Df that have the potential to significantly improve disease severity in individuals with this allele or other alleles that retain significant function.

Recently, there have been efforts to identify and develop TPI Df therapies (Vogt et al., 2021; Vollmer et al., 2025). Human cellular models of TPI Df have been developed using the common *TPI^E105D^* allele fused to a fluorescent protein gene that enables image-based drug screening (Vogt et al., 2021). Additionally, stable cells expressing TPI^E105D^::GFP and a reactive oxygen species biosensor have been developed into a high-throughput screen to identify new compounds capable of increasing TPI to therapeutically relevant levels without significant toxicity (Vollmer et al., 2025). Among the lead compounds identified by this group was a 1,2,4-triazine (Molport 002–877–424) a molecule reported to be a hypoxia inducible factor (HIF) activator. Compound 424 increases TPI levels significantly in both *TPI^E105D^* and *TPI^R5G/f.s.^* cells, suggesting that HIF activation may be a viable mechanism of action for TPI Df therapy (Figura et al., 2025; Vollmer et al., 2025).

Prolyl hydroxylase domain (PHD) inhibitors (PHDIs) are a recently developed class of small molecules that induce HIF. HIF is a heterodimer consisting of an oxygen sensing alpha subunit, HIF-1α, 2α or 3α, and the primary regulatory subunit, HIF-β. There are three isoforms of HIF PHD (PHD1, PHD2 and PHD3). These enzymes are 2-oxoglutarate-dependent dioxygenases that hydroxylate HIF-1α in an iron-dependent manner under normoxic conditions, targeting HIF-1α to the proteasome for degradation (Hon et al., 2002; Jaakkola et al., 2001; McNeill et al., 2002; Schofield and Ratcliffe, 2004). Under hypoxic conditions, or with pharmacological inhibition, PHDs are inhibited and HIF-1α is stabilized. HIF-1α translocates to the nucleus where it dimerizes with HIF-β to form an active HIF transcription complex. This complex binds to hypoxia-response elements (HREs) found in the promoter regions of target genes, causing the transcription and upregulation of many genes essential for regulatory processes (reviewed by Zhang et al., 2025). There are numerous known target genes of HIF-1α, many of which are involved in glycolysis, metabolic reprogramming, and erythropoiesis (Benita et al., 2009; Dengler et al., 2014). Currently, there are four PHD inhibitors that are in different phases of clinical development for treatment of anemia in individuals with chronic kidney disease (CKD): FG4592 [i.e. roxadustat, Kyntra Bio (formerly FibroGen)], GSK1278863 (i.e. daprodustat, GSK), AKB-6548 (i.e. vadadustat, Akebia Therapeutics) and Bay85-3934 (i.e. molidustat, Bayer). These compounds were developed and primarily studied as treatments for anemia caused by CKD (Yeh et al., 2017). In the context of CKD, the mechanism of action appears to primarily be HIF activation leading directly to increased expression of the erythropoietin (*EPO*) gene. As HIF activation has also been shown to also activate TPI expression (Benita et al., 2009), we sought to examine whether currently available PHDIs would function in TPI Df.

Here, we report a high-resolution crystal structure of TPI^R5G^ and the effect of multiple PHD inhibitors on TPI^R5G^ protein levels, using previously established human *TPI^R5G/f.s.^* cells (FB909). FB909 cells have very low TPI protein levels, consistent with the TPI^R5G^ protein having poor cellular stability. Treatment with various PHD inhibitors significantly increased expression of the mutant protein. Additionally, TPI activity levels significantly increased after PHD inhibitor treatment. The data support the conclusion that PHDIs have the potential to significantly mitigate TPI Df disease and should be further investigated and developed as a therapy.

## RESULTS

**Fig. 1.**
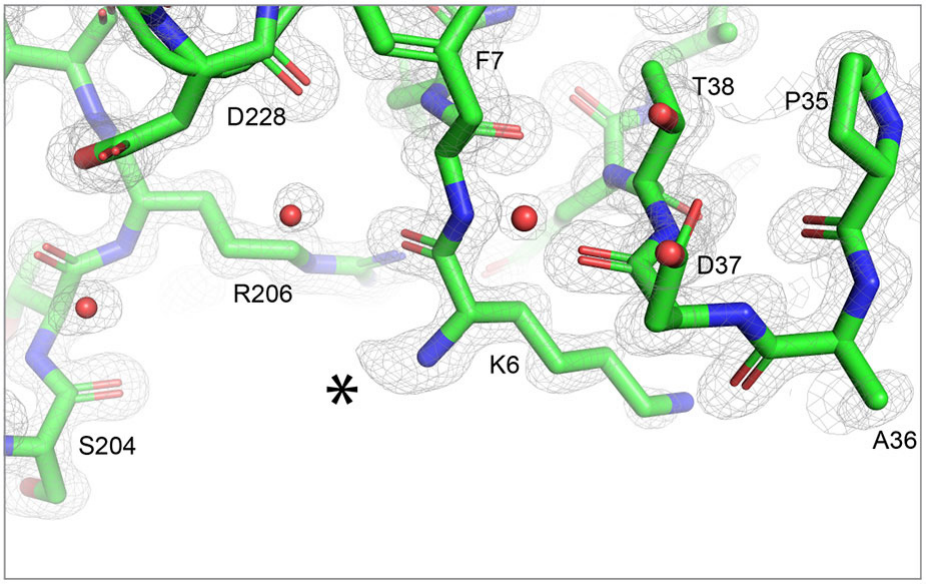
**The R5G mutant destabilizes the N terminus of TPI.** Electron density in the vicinity of R5G. TPI protein model in this region shown as sticks (green). Electron density (gray mesh) from a 2Fo-Fc composite omit map contoured at 1.2 σ. The approximate location of G5 (which is disordered) is indicated by an asterisk.

To better understand the physical basis for TPI Df resulting specifically from the *TPI^R5G^* disease-causing variant, we crystallized and determined the structure of human TPI^R5G^. The crystals contain both subunits of the TPI dimer within the asymmetric unit and the structural model was refined to R_work_/R_free_ values of 12.4/13.8% at a resolution of 1.15 Å. As with previous structures of TPI (Roland et al., 2013, 2015, 2016, 2019; VanDemark et al., 2022), clear electron density for a phosphate ion believed to have co-purified with the protein was found within the active site of subunit B, and a nearby bromide ion from the crystallization conditions was observed occupying the sugar-binding portion of the active site pocket. Electron density for the entirety of both subunits was observed starting at lysine 6. As depicted in Fig. 1, a small portion of electron density corresponding to the TPI Df-causing glycine (R5G) was observed; however, the observed density does not encompass the entire glycine residue and thus could not confidently be built. There was no density at all corresponding to residues 1-4 (Fig. 1).

**Fig. 2.**
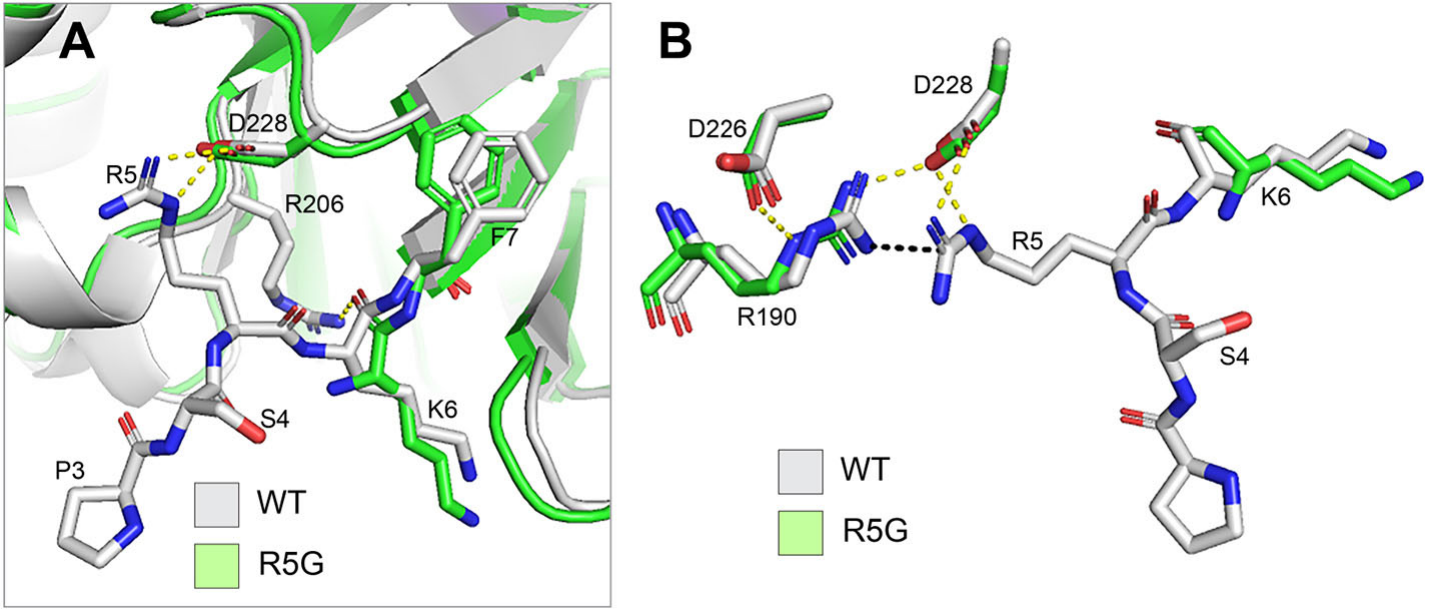
**Changes in TPI structure resulting from the R5G mutation.** (A) The TPI WT (PDB: 4POC, white) and R5G (green) structures were superposed and visualized in the region surrounding R5G. (B) Positioning of residues in the R5/R190/D226/D228 electrostatic network in both the WT (white) and R5G (green) structures. Hydrogen bonding and salt bridge interactions are shown as yellow dashed lines. A van der Waals interaction between R190 and R5 in WT TPI is shown as a black dashed line.

To analyze the difference in TPI structure resulting from the R5G substitution, we aligned the TPI^R5G^ structure with the wild-type (WT) TPI structure from PDB 4POC, which was crystallized in the same conditions. As mentioned above, residues P3-S4-R5 were not observed in the electron density of the R5G structure (Fig. 2A), although they are commonly observed in a variety of TPI structures (Roland et al., 2013, 2015, 2016, 2019; VanDemark et al., 2022). In the TPI^WT^ structure, R5 has been observed making important contacts, prominently a salt bridge with D228. Nearby residue D226 and D228 have been shown previously to be participating in stabilizing interactions between the seventh and eighth segment of the beta barrel with the rest of the fold (Roland et al., 2019). Further, R5 hydrogen bonds directly with the sidechain of R190. *TPI^R190Q^* is a previously identified TPI Df pathogenic mutation in which the mutant protein was shown to exhibit increased TPI turnover in human cells, neurologic dysfunction in *Drosophila*, and changes in the coordination between the substrate binding site and catalytic residues in the human protein (Roland et al., 2019). Thus, R5 is positioned within a network of electrostatic interactions that is important for enzyme activity and protein stability (Fig. 2B). In the TPI^R5G^ structure, it is interesting that the positions of residues D226, D228 and R190 are largely similar to the those in TPI^WT^, suggesting that R5 is not a ‘keystone’ type of residue that sets the foundation for positioning the rest of this electrostatic network. Changes to residues in this network have been shown to reduce TPI catalytic activity (Silverman et al., 2001); however, this is not the case for TPI^R5G^ (Figura et al., 2025), further supporting that the impact of the R5G substitution does not lie within its potential to alter enzyme activity, stoichiometry or structural organization of the enzyme.

### PHDIs increase TPI protein levels

**Fig. 3.**
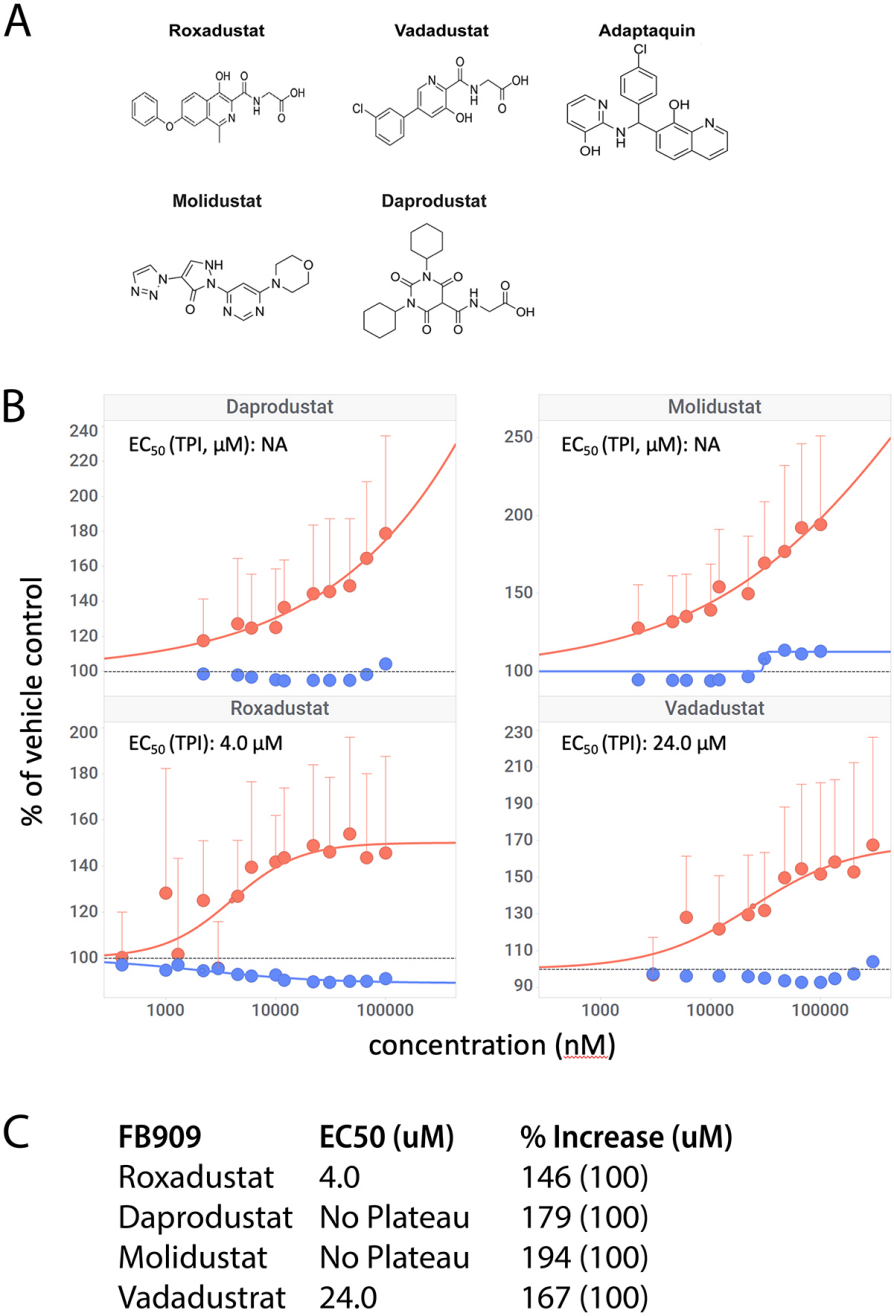
**PHD inhibitors increase TPI in FB909 fibroblasts. (**A) Structures of HIF activators/PHDIs. **(**B) Ten-point dose responses showing increases in TPI in FB909 cells (red; R5G/f.s.). Nucleus size is shown in blue, which suggests the compounds are not toxic at these doses. All four PHDIs appear to be active. (C) EC50s and maximum increases for TPI are shown for the compounds. In some cases, the curves did not plateau so an EC50 could not be calculated. Values in parentheses are the concentrations at which the TPI response was maximal. Error bars represent s.d.

Previously, we developed a high-content screening platform to identify potential TPI Df therapies and screened ∼225,000 compounds (Vollmer et al., 2025). Several promising compounds were identified that increased TPI protein levels including several compounds that had previously been reported as HIF activators. These findings suggested that other classes of HIF activators may prove functional for TPI Df. An emerging class of HIF activators are the PHDIs. We examined several PHDI/HIF activators for efficacy in FB909 cells: roxadustat, molidustat, daprodustat, vadadustat and adaptaquin (Fig. 3A).

Initially, to investigate the effect of PHDIs on TPI expression in FB909 fibroblasts (derived from individuals with TPI Df), we performed a ten-point dose response using an established immunohistochemical method with four PHDIs whereby TPI levels and nucleus size are used to determine efficacy as well as toxicity (Fig. 3B). The results showed that all four PHDIs examined resulted in dose-dependent increases in TPI levels without corresponding decreases in nucleus size (Fig. 3B). The magnitude of the observed increases in TPI and, where a sigmoidal curve was obtained, EC50s were calculated (Fig. 3C).

**Fig. 4.**
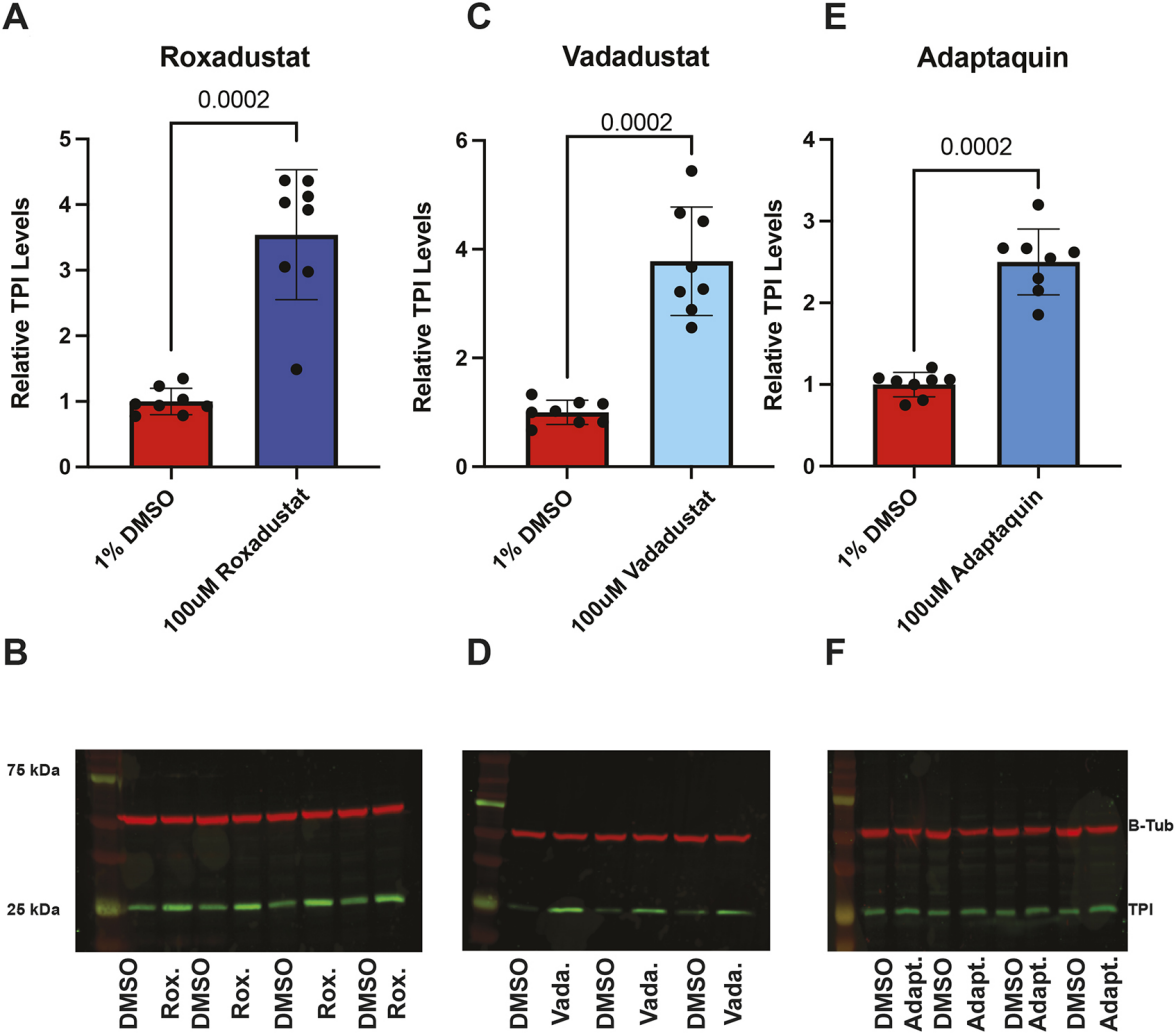
**PHD inhibitor treatment of FB909 cells.** (A) Western blot analysis demonstrates an average ∼354% increase in relative TPI protein levels after treatment with 100 µM roxadustat compared to DMSO vehicle controls. (B) Representative blot used for analysis of 100 µM roxadustat treatment. (C) Vadadustat western blot analysis shows a significant average increase of ∼378% compared to vehicle controls. (D) Representative vadadustat blot used for analysis of 100 µM vadadustat treatment. (E) Adaptaquin analysis shows a significant increase of ∼250% in relative TPI levels compared to DMSO controls. (F) Representative adaptaquin blot used in 100 μM adaptaquin treatment analysis. Two-tailed Mann–Whitney tests were used to determine significance. *P*-values are displayed; *n*=8 for each condition; error bars represent s.d.

To confirm the immunohistochemical results, we performed western blot assays to measure relative TPI protein levels after treatment with two PHD inhibitors, roxadustat and vadadustat (Fig. 4A-D). These data reveal that roxadustat (100 µM) and vadadustat (100 µM) significantly increased relative TPI protein levels compared to vehicle-treated controls by ∼350 and ∼375%, respectively (Fig. 4A-D). We then examined whether a newer, less-studied PHDI/HIF activator, adaptaquin, also functioned to increase TPI levels in FB909 cells. As with roxadustat and vadadustat, adapataquin (100 µM) significantly increased TPI relative to vehicle controls (Fig. 4E,F). The increase in TPI protein observed with adaptaquin was ∼250%.

### Vadadustat increases TPI activity in FB909 cells

**Fig. 5.**
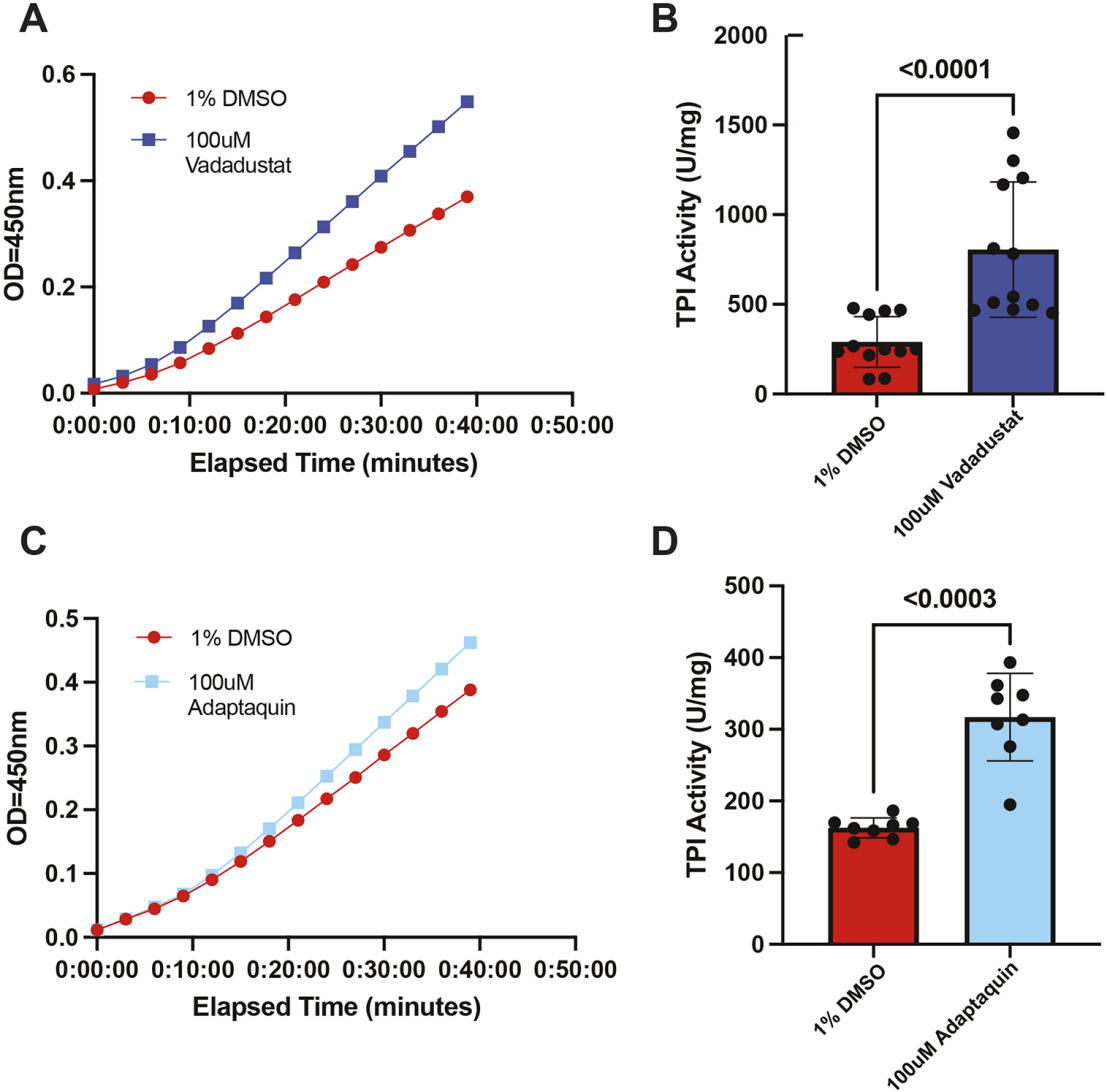
**PHDIs increase TPI enzyme activity.** (A) Representative kinetic graph demonstrating changes in background corrected absorbance in 100 μM vadadustat-treated samples compared to controls. (B) TPI activity analysis showing a significant increase in catalytic activity in 100 µM vadadustat-treated samples compared to DMSO controls. Two-tailed Mann–Whitney test was used to determine significance. *P*-values are reflected above; *n*=12. (C) Representative graph representing kinetic changes in absorbance between 100 µM adaptaquin-treated samples compared to DMSO controls. (D) TPI activity analysis demonstrating a significant increase in catalytic activity in 100 µM adaptaquin-treated lysates compared to controls. Two-tailed Mann–Whitney test was used to determinate significance. *P*-values are shown; *n*=8; error bars represent s.d. OD, optical density.

As treatment with 100 µM vadadustat produced the largest increase in TPI levels from western blot analysis, we initially measured how this compound would affect TPI activity in FB909 cells. Cells were treated with 100 µM vadadustat for 48 h, harvested, and lysed to measure the total cellular activity of expressed TPI proteins. Prior to activity analysis, lysate samples were split to calculate total protein levels using a bicinchoninic acid (BCA) assay to continue protein activity assays. Treatment with 100 µM vadadustat significantly increased TPI activity compared to vehicle control (Fig. 5A,B). Raw absorbance data were normalized to total cellular protein to generate TPI protein activity levels, in U/mg, as described in the Materials and Methods. We similarly examined adaptaquin for an effect on cellular TPI activity. Adaptaquin (100 µM) also produced a significant increase in cellular TPI activity over DMSO-treated controls (Fig. 5C,D).

### Therapeutically relevant increases?

**Fig. 6.**
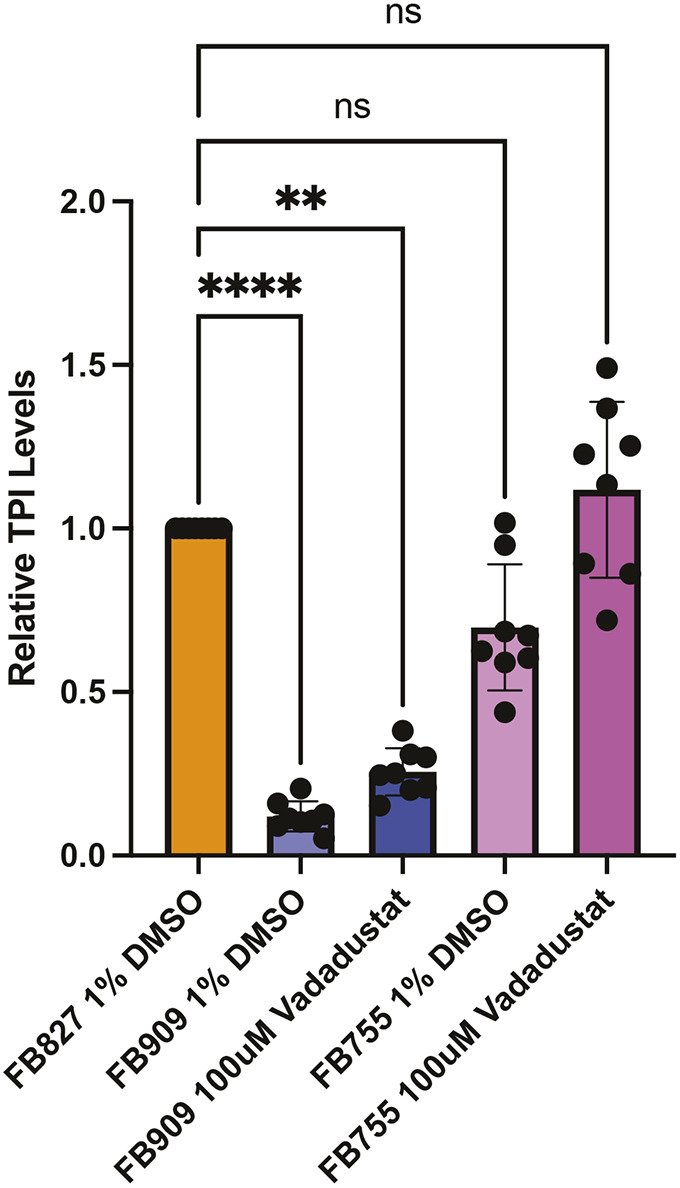
**Vadadustat treatment of multiple cell lines compared to WT fibroblasts.** (A) Western blot analysis shows a ∼2-fold increase in relative TPI levels with vadadustat treatment of FB909 cells compared to DMSO controls. This increase was enough to make the vadadustat-treated FB909 samples less significantly different in comparison to WT levels. Kruskal–Wallis nonparametric statistical test was used to evaluate significance. ****P<0.00001, **P<0.001; *n*=8; error bars represent s.d.

The increases seen in TPI protein levels following PHD inhibitor treatment led us to investigate whether TPI in treated FB909 cells would increase to the levels seen in cells from a less-affected, compound heterozygous individual (FB755; Q181P/E105D) or possibly to levels in WT fibroblasts (FB827). We conducted western blot assays comparing TPI levels in FB909 (*TPI^R5G/f.s^*) to FB755 (*TPI^Q181P/E105D^*) cells treated with 100 µM vadadustat or vehicle (1% DMSO) compared to FB827 (WT) cells. As before, we saw a highly significant increase in FB909 TPI protein levels after vadadustat treatment; however, this increase was not enough to bring protein levels up to that of WT or even to that of untreated FB755 cells. These results are consistent with previous findings that FB909 cells exhibit an ∼90% reduction in TPI protein compared to WT fibroblasts due to the lack of protein from the frame-shift mutation and the low stability of the TPI^R5G^ protein (Figura et al., 2025). One would predict that a 90% reduction followed by a 378% increase might result in levels that are∼38% of normal, which is approximately what was observed (Fig. 6). Further studies will be needed to know the extent to which protein levels increase significantly in relevant tissue types with PHDI treatment and whether these changes would be beneficial *in vivo*.

### Investigating other disease targets

To investigate whether the increases in TPI we observed were the result of transcriptional upregulation of *TPI1* or a secondary effect of a protein that altered the stability of TPI, we performed an RNA-sequencing (RNAseq) experiment. FB303 and FB909 TPI Df cell lines were studied to compare the regulation of genes after a 48-h treatment with 100 µM vadadustat and were compared to vehicle-treated controls. The experiment sequenced more than 200 million reads, totaling 20-30 million read pairs, from three independent RNA samples from each group (FB909 vadadustat, FB909 DMSO, FB303 vadadustat, FB303 DMSO), with ∼500 loci exhibiting significant differences between the treated cells and their DMSO controls. There were 493 significantly differentially expressed genes identified when comparing FB303 vadadustat-treated samples with their DMSO controls, with 370 upregulated genes and 123 downregulated genes being identified. In the FB909 vadadustat samples, there were 439 significantly differentially expressed genes identified compared to DMSO controls, with 383 upregulated and 56 downregulated genes. The complete raw data sets, gene ontology reports, all identified upregulated genes, and mapping statistics are provided (Table S1). Our analyses of the RNAseq data show at least 23 known HIF target genes are upregulated with vadadustat treatment in both FB909 and FB303 cells, consistent with the known mechanism of action of vadadustat via HIF activation (Table S2). Additionally, TPI was significantly increased in FB303 cells (1.48 log2 fold, adjusted *P*<0.01) and in FB909 cells consistent with the increase we observed in protein levels (Table S3). These data support the conclusion that HIF activation resulting from vadadustat treatment is directly increasing the expression of TPI that leads to the steady-state protein levels we observed. As increased expression of mutant TPI leads to increases in TPI protein and activity, this should be therapeutically beneficial.

We then examined whether other genes that exhibited significant increases in expression with HIF activation might be disease associated and likewise be predicted to benefit from HIF activation therapy. Such diseases would be caused by hypomorphic, loss-of-function or protein stability mutations. Such mutations would still produce proteins with function and, thus, increasing the expression of these proteins would be therapeutically beneficial. In total, we identified 27 additional loci with significantly increased expression (cutoff used was an adjusted *P*-value of <0.01 in both cell lines) that have known or predicted hypomorphic loss-of-function or stability mutations that are disease associated (Table S3). It should be noted that these are hypothesis-generating observations identified in the robust results obtained through RNAseq, and require additional study and functional validation.

### HIF antisense negative feedback regulation

**Fig. 7.**
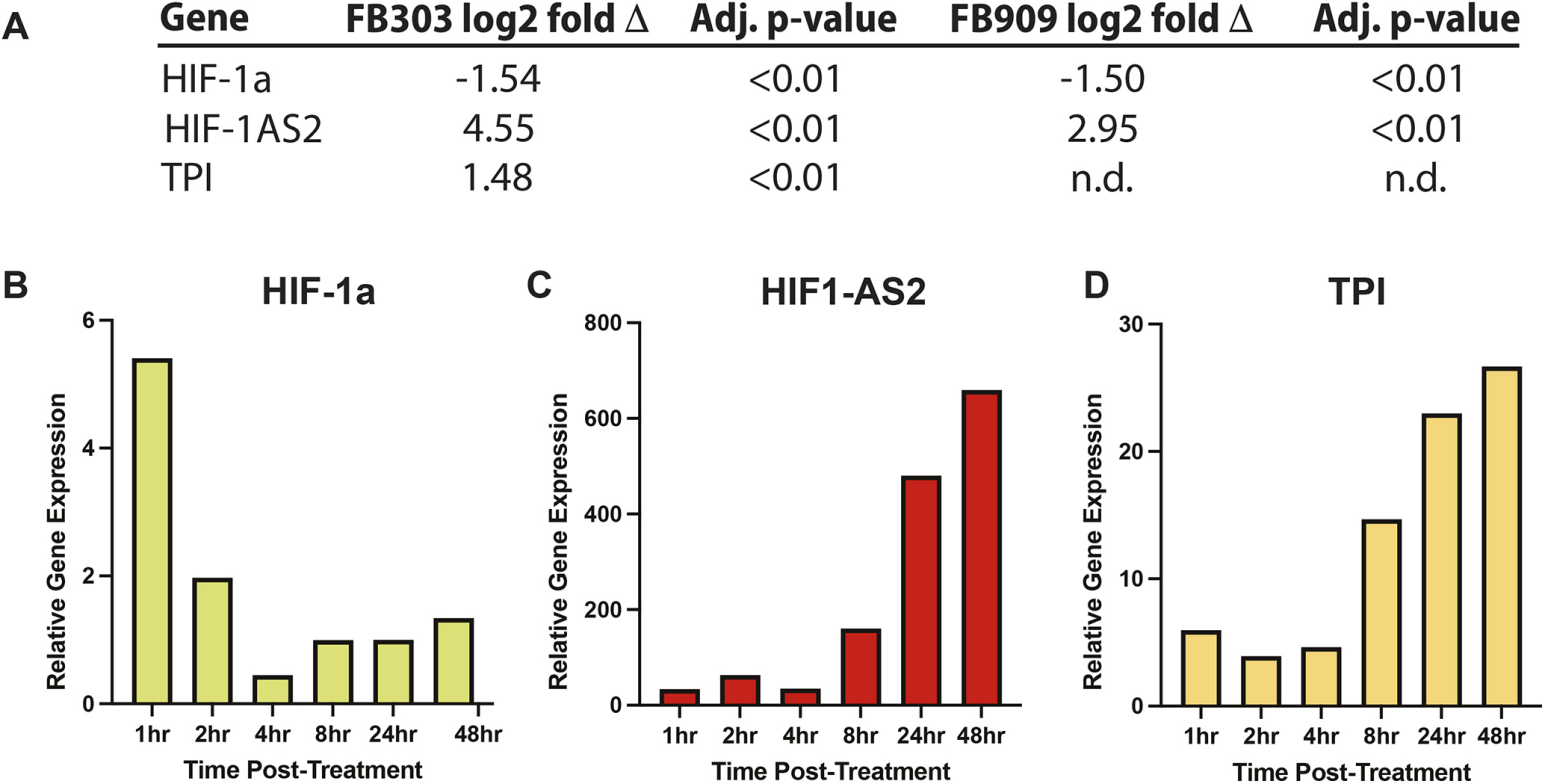
**Chronic PHDI treatment activates HIF antisense negative-feedback regulation.** (A) Treatment of FB303 with 100 µM vadadustat (48 h) resulted in an increase in *TPI* expression, relative to vehicle-treated control cells. Interestingly, we noted a marked increase in *HIF-antisense 2* expression (*HIF1-AS2*) and a decrease in *HIF1A*. Similar results were obtained with FB909 cells for *HIF1A* and *HIF1-AS2*; however, basal expression of *TPI* in the vehicle-treated cells was too low to quantify the fold increase with vadadustat treatment (data not shown). *n*=3, all groups. (B-D) RT-qPCR was used with WT fibroblasts (FB826) to verify the changes observed by RNAseq, confirm them in the context of WT cells, and to determine the timing of the attenuated HIF response. As in A, 100 µM vadadustat treatment was used with varying length of exposure. *HIF1A* expression decreased and *HIF1-AS2* expression increased markedly with prolonged vadadustat exposure. *TPI* expression increased with vadadustat treatment and remained high, suggesting there is sustained activation with long-term exposure.

In FB303 cells, *TPI* and HIF1-antisense 2 (*HIF1-AS2*) were identified as highly upregulated and significant; however, *HIF1A* expression was downregulated. Similarly, in FB909 cells *HIF1-AS2* was upregulated, while *HIF1A* was significantly downregulated (Fig. 7A). TPI expression was apparently upregulated in the FB909 with vadadustat treatment; however, the effect could not be quantified due to the low basal expression of TPI in vehicle-treated cells (data not shown). Based on the RNAseq results, we wanted to further investigate the changes in *HIF1A* and *HIF1-AS2* after treatment with a PHD inhibitor, and how this affects target genes of HIF-1α (like *TPI*). We also wanted to gain a more specific understanding of the timing of the HIF response after 100 µM vadadustat treatment. We harvested vadadustat-treated samples after 1, 2, 4, 8, 24 and 48 h. After RNA isolation and purification and reverse transcription, we performed quantitative PCR (for primer sequences see Table S4). The reverse transcription quantitative polymerase chain reaction (RT-qPCR) results informed us that *HIF1A* was at peak expression after 1 h of vadadustat treatment, then steadily decreased with longer drug exposures. Alternatively, *HIF1-AS2* expression steadily increased with drug exposure up to the 48-h time point. *TPI* also steadily increased up to the 48-h time point (Fig. 7B). This signifies that there is activation of *TPI* even with long-term PHDI treatment.

## DISCUSSION

TPI Df is a devastating ultra-rare childhood disease characterized by neuromuscular impairment and anemia, typically leading to early death. There are currently no treatments for this disease outside of supportive care or dietary changes, such as the ketogenic diet. The ‘common’ mutation *TPI^E105D^* is the most frequently identified mutation, and individuals can be homozygous or have the combination of another allele that can either worsen disease or cause them to have lower symptom severity and prolonged lifespan. We have previously reported an individual with two novel alleles, *TPI^R5G/f.s.^*, which presents as a form of slow-progressing TPI Df with severe neuromuscular symptoms but lacking anemia (Figura et al., 2025). *TPI^R5G/f.s.^* cells (FB909) exhibit a ∼90% reduction in TPI protein levels compared to WT cells, and, although the *TPI^R5G^* allele has low steady-state levels, it remains catalytically active (Figura et al., 2025). In our previous search for a potential therapeutic using high-throughput screening techniques, we identified HIF activators as a lead class of compounds for TPI Df treatment (Vollmer et al., 2025). PHDIs have primarily been studied for their ability to treat CKD with anemia because of their ability to upregulate the *EPO* gene. HIF activation is known to upregulate numerous target genes, many of which are involved in energy metabolism (Benita et al., 2009; Zhang et al., 2025). Here, we discovered that PHDIs induce many of the known HIF target genes, including TPI, making them potentially valuable to repurpose for TPI Df therapies.

Numerous previous structural studies have demonstrated a conserved interaction between R5 and a network of salt bridges and electrostatic interactions that impact enzyme activity and cellular function. Here, we found in the high-resolution structure of TPI^R5G^ that the loss of the arginine side chain did not alter the positioning of this electrostatic network in any significant manner. The loss of the interaction between R5 and R190 and D228 did destabilize the position of the mutated residue, G5, and this in turn imparted flexibility into loop 1 of TPI (residues 1-5), all of which are disordered in the R5G structure presented here. In cells, the R5G mutation results in a significant increase in protein turnover in cellular models of TPI Df, suggesting there is a mechanism driving cellular turnover that is activated by the R5G substitution, such as enhanced engagement by protein quality-control machinery. It is also noted that the R5G mutant confers enhanced thermal stability for the mutant protein (Figura et al., 2025), a characteristic that has also been observed in several other pathogenic mutant proteins, suggesting there is a disconnect between overall thermal stability and mutant protein stability that is hypothesized to be mediated by quality-control machinery. This further suggests that small regions of the protein could be the target recognized by this machinery. It is plausible therefore that the loop 1 region could be playing this role in TPI^R5G^. Alternatively, the N terminus could be required for other hypothesized ‘moonlighting’ functions of TPI and it is loss of these functions that lead to an increase in protein turnover (Myers and Palladino, 2023).

Whereas our studies primarily aimed to provide proof of concept to support a role for PHD inhibition in increasing levels of mutant TPI, our *in vitro* studies in cells from individuals with the TPI Df support repurposing PHDIs for potential treatment of this disease. We found that vadadustat, roxadustat, and adaptaquin were all successful at significantly increasing TPI protein levels in cells from individuals with TPI Df. To determine if the increases observed could be therapeutically valuable, we compared vadadustat treated FB909s with a WT cell line (FB827) and cells from a compound heterozygote cells from an individual with TPI Df (Q181P/E105D) with a less-severe disease course (FB755) treated with vadadustat and DMSO controls. While the protein levels of FB909's were significantly increased by ∼378%, this was not enough to bring the levels up to that of WT cells. The decrease in TPI observed in FB909 cells was marked (90% reduced), which would be difficult to overcome. Additionally, one of the alleles is a predicted null, so transcriptional activation only increases protein from one of the two alleles. Thus, it is not clear whether this dramatic increase in TPI would be sufficient to ameliorate disease symptoms in an individual with the *TPI^R5G/f.s.^*genotype. Our immunofluorescence results show EC50s of 4 µM and 24 µM for roxadustat and vadadustat, respectively. In rats, a single dose of vadadustat reached mean C_max_ values of 50,900 ng/ml and 62,800 ng/ml in males and females, respectively, which correlate to plasma levels of∼165-200 µM (Zuk et al., 2022). C_max_ data in humans are similar with reported plasma levels of ∼165 µM (FDA_Access_Data, 2024). While our data show TPI-inducing activity at drug levels below what is achievable in human individuals, further *in vivo* testing is needed to demonstrate pre-clinical efficacy. We also conducted TPI activity assays that demonstrate that PHDI treatment (adaptaquin and vadadustat) significantly increases cellular activity of the TPI enzyme. The TPI cellular activity increases observed with PHDI treatment are consistent with the increases in TPI protein observed by western blot.

PHDIs have a known mechanism of action involving HIF transcriptional activation. To investigate transcriptional upregulation with PHDI treatment, we conducted RNAseq experiments in two distinct TPI Df cell types treated with 100 µM vadadustat. We identified 23 known HIF targets that were upregulated in both cell lines, consistent with the established mechanism of action of PHDIs. We also identified significant increases in *TPI1* mRNA, consistent with the TPI protein increases observed with PHDI treatment. Results from the RNAseq experiments also showed significantly increased expression of 27 disease-associated genes that are caused by hypomorphic or haploinsufficient loss-of-function mutations, all of which could potentially benefit from treatment with a HIF activator. Additional studies investigating whether PHDI treatment that leads to increased expression of these alleles results in increased protein activity would help determine which diseases would benefit from PHDI repurposing.

Interestingly, HIF-1α expression was significantly downregulated over time, but HIF1-AS2 was highly upregulated. This is a known negative-feedback mechanism that serves to attenuate HIF1 transcriptional activity (Chen et al., 2017; Zhong et al., 2024). The downregulation of HIF-1α could potentially be multifactorial, but for our purposes we decided to look more closely at the changes between *HIF1A* and *HIF1-AS2* mRNA levels. We evaluated these changes in *HIF1A* and *HIF1-AS2* expression by RT-qPCR, and identified that *HIF1A* expression is at its peak ∼1 h post-PHDI treatment and declines with chronic vadadustat exposure. By contrast, *HIF1-AS2* steadily increased expression over the time course studied (48 h). *TPI1* mRNA expression showed similar results, increasing steadily over the time course studied. These data suggest that a HIF inactivation feedback loop is activated by chronic vadadustat treatment. These data, while not conclusive, demonstrate a need for gaining a deeper understanding of HIF1-AS2 and how to reset this HIF-1α/HIF1-AS2 feedback loop to maintain HIF-1α activation. Additional studies with other PHDIs and in other tissues or cell types are needed to better appreciate the implications the activation of this feedback loop has on dosing strategies and how this affects HIF-1α upregulation.

### Conclusion

The data reported here suggest that repurposing of PHDIs for TPI Df treatment is a promising strategy. The crystal structure of TPI^R5G^ demonstrates the protein is structurally normal aside from the very N terminus (residues 1–5), suggesting this is sufficient to trigger a reduction in stability that leads to markedly reduced TPI steady-state levels. Lastly, chronic PHDI treatment leads to the activation of a HIF-1α/HIF1-AS2 feedback loop, which has implications for dosing and treatment of chronic diseases for which sustained HIF activation is needed.

## MATERIALS AND METHODS

### Cloning, expression and purification

The coding sequences of human *TPI1* WT and R5G mutant were cloned into pLC3 encoding TPI with a His_6_-MBP tag in the N terminus that is cleavable by TEV protease. Protein expression was performed in BL21 (DE3) CodonPlus-RIPL *Escherichia coli* cells. Cell cultures were grown in LB at room temperature for 16-18 h and induced through the addition of 0.2 mM isopropyl β-D-1-thiogalactopyranoside (IPTG). Cells were harvested by centrifugation (5 min at 10,000 ***g***) and lysed by homogenization with buffer conditions of 25 mM Tris-HCl pH 8, 500 mM NaCl, 10% glycerol, 5 mM imidazole and 1 mM 2-mercaptoethanol. Cellular debris was removed by centrifugation at 30,000 ***g*** for 30 min. His-MBP-TPI fusion protein was purified using nickel-affinity chromatography, followed by TEV protease digestion to separate the His₆-MBP tag from TPI. A second round of nickel-affinity chromatography was performed, followed by anion-exchange chromatography (HiTrap-Q) and size-exclusion chromatography. The peak protein fractions were concentrated to 6-8 mg/ml in 20 mM Tris-HCl pH 8.8, 200 mM NaCl and 1 mM 2-mercaptoethanol and stored at 4°C for biochemical assays and crystallization. The purity was >99% as verified by SDS-PAGE.

### Crystallization and structural determination

Crystals of hTPI^R5G^ were grown using the sitting-drop vapor diffusion method at 20°C. The crystals grow over 2 days in a drop containing 1 μl of TPI^R5G^ at 7.5 mg/ml and 2 μl of well solution containing 24-25% polyethylene glycol 2000 monomethyl ether (MME; Spectrum Chemical) and 50 mM potassium bromide, as in previous structural studies. The crystals were cryoprotected in 40% polyethylene glycol 2000 MME and 50 mM potassium bromide and flash-frozen in liquid nitrogen prior to X-ray diffraction. Diffraction data were collected at APS and processed and scaled using autoPROC (Vonrhein et al., 2011) using the standard isotropic scaling regime, with I/σI>0.0 and CC(1/2)>0.3 as cutoffs. Crystals belong to the space group P2_1_2_1_2_1_ with *a=* 65.2, *b=*73.0, *c=*93.0 Å. Initial phases were estimated using molecular replacement with Phenix (Liebschner et al., 2019) using TPI^WT^ (PDB: 4POC) as a search model. The resulting model was then improved through rounds of positional refinement within COOT (Emsley et al., 2010) and anisotropic B-factor refinement. Model quality was validated using MolProbity tools in Phenix (Davis et al., 2007). Crystal statistics are provided in Table S5.

### Cell culture

FB909 fibroblasts were previously published (Figura et al., 2025). Standard sequencing methods were used to confirm the genotype of FB909 cells (R5G, frameshift mutation) (Figura et al., 2025). FB755, FB303 and FB827 fibroblasts were all previously reported (VanDemark et al., 2022; Vogt et al., 2021; Vollmer et al., 2025). Fibroblasts were cultured following standard incubation procedures (37°C, 5% CO_2_) in complete media containing 20% defined fetal bovine serum (FBS) (Cytiva, SH3007003), Dulbecco's Modified Eagle Medium (DMEM) (Corning), 2 mM L-glutamine (Gibco, 25030081), 100 U penicillin/100 μg streptomycin/ml (Sigma-Aldrich, P4333-100ML) and non-essential amino acids (Cytiva, SH3023801). For experiments, 10% FBS DMEM media was used. Cell media was replenished every 2-3 days, and, once confluent, cells were passaged following previously reported methods (Figura et al., 2025).

### Cell counting and drug treatment

Cells were counted and plated for experiments following previously reported methods (Vollmer et al., 2025). After fibroblasts were plated and allowed to adhere to a cell culture dish for 24 h, the medium was aspirated and replaced with fresh medium containing either vadadustat (Akebia Therapeutics), molidustat (Selleckchem, S8138) daprodustat (Selleckchem, S8171), roxadustat (AK Scientific, J96601), adaptaquin (MedChem Express, HY-101449) or DMSO (Sigma-Aldrich, D4540-500ML) as a vehicle control. Treatment solutions were prepared by diluting compound stocks in DMEM with 10% FBS. Plates were left with 10 ml of each solution for 48 h and incubated under standard culture conditions. After 48 h of treatment, cells were trypsinized, pelleted, washed with PBS, and resuspended in RIPA buffer with protease inhibitors [PMSF (100 μM), Pepstatin A (0.5 μg/μl) and leupeptin (1 μg/μl)]. Cells were lysed using pulse sonication, and protein concentrations were analyzed using a BCA assay (Pierce, 23225).

### Immunofluorescence in TPI-Df cells

Ten-point dose responses were performed as previously reported (Vollmer et al., 2025). Briefly, attached fibroblasts in 384-well Phenoplates (Revvity) were treated with test agents in ten-point, two-fold compound gradients for 48 h, fixed with 4% formaldehyde, and processed for imaging. Cells were stained with 10 μg/ml Hoechst 33342, 1:1000 rabbit polyclonal TPI1 antibody (PA5-21583, WH3264660A, Invitrogen) and 1:500 Cy3-conjugated AffiniPure Donkey Anti-Rabbit IgG (H+L) secondary antibody (Jackson ImmunoResearch, 711-165-152). Plates were imaged on an OPERA Phenix Plus high content reader (Revvity) with a 20× air objective, laser lines, and emission filters for Hoechst (ex405/em435-480 nm) and Cy3 (ex561/em570-630 nm). Images were analyzed and archived in Harmony 5.2.

### Western blotting

Western blot methods detailing SDS-PAGE sample preparation, semi-dry protein transfer, primary and secondary antibody incubation, blocking, washing and imaging steps were previously reported (Figura et al., 2025). Samples were heat-denatured at 74°C for 5 min before being loaded onto a 12% SDS-PAGE gel, and 15 μg of protein was loaded from each sample. The primary antibody solutions consisted of anti-TPI (1:5000, rabbit polyclonal; Invitrogen, PIPA521583) and anti-β-tubulin (1:1000, mouse polyclonal; Developmental Studies Hybridoma Bank, E7-c) diluted in Intercept PBS Blocking Buffer (LI-COR BioTech LLC, NC1660556). Following overnight primary antibody incubation, blots were washed in phosphate buffered saline with Tween 20 (PBS-T), and incubated in the dark with secondary antibody solution for 2 h. The secondary antibody solution contained 10 ml Intercept PBS Blocking Buffer, 10 μl Tween 20, goat-anti-mouse 680 (1:20,000; Fisher Scientific, A21121) and donkey-anti-rabbit 800 (1:20,000; Fisher Scientific, NC9523609). After secondary incubation, blots were washed in PBS-T six more times for 5 min each. The blots were then imaged and analyzed using a LI-COR Odessey CLX (CLX-2346) machine. Uncropped images from this paper are shown in Fig. S1.

### TPI enzymatic activity assay

Cells were plated in 10 cm dishes, allowed to adhere overnight, and then treated as described below. A 100 µM solution of vadadustat or adaptaquin was made by diluting 100 µl of a 10 mM stock into 9.9 ml of DMEM with 10% FBS. A vehicle control containing 1% DMSO in 10% FBS DMEM was prepared in parallel. After aspirating the medium, cells were treated for 48 h, then harvested following the procedure described in the ‘Cell culture’ section.

The TPI activity assay was then performed following previously reported methods (Figura et al., 2025). Absorbance of drug-treated lysate samples, standards and positive controls were recorded at 450 nm every 3 min for 40 min at 37°C. Data were normalized to protein concentration and converted to NADH concentrations using the standard curve. TPI activity was calculated according to the kit protocol, using two time points within the linear phase of the reaction to determine slope values. Activity was expressed in U/mg of protein.

### RNAseq

To prepare FB909 fibroblasts for RNAseq, cells were plated at a seeding density of 100,000 cells per 100 mm×20 mm dish. After a 24-h adherence period, cells were treated in triplicate with 1% DMSO and 100 µM vadadustat. Drug-treated plates were placed back in the cell culture incubator for 48 h, and harvested following methods mentioned in the ‘Cell counting and drug treatment’ section. Following the last centrifugation step of the cell harvest, the supernatant was aspirated and cell pellets were snap-frozen in liquid nitrogen. Samples were sent to Azenta Life Sciences for RNAseq analysis. Genes that were identified as having significantly increased expression in both cell lines with vadadustat treatment were searched in Online Mendelian Inheritance in Man (OMIM; https://www.omim.org) for genetic disease association and the mechanism inheritance was investigated to identify those for which an increase in expression could be beneficial.

### RT-qPCR

FB826 cells were treated with 100 µM vadadustat and a 1% DMSO vehicle control, and harvested at different time points. For the vehicle control, we treated FB826 fibroblasts with 1% DMSO for 8 h before cell harvest. Vadadustat samples were harvested at the following time points: 1 h, 2 h, 4 h, 8 h, 24 h and 48 h. At the end of cell harvesting, cell pellets were resuspended in 500 µl of TRI reagent (Fisher, R2050-1-200) to lyse the samples to isolate RNA. To purify RNA, the Direct-zol RNA MiniPrep Plus kit (Fisher, R2070) was used. RNA was quantified using a NanoDrop spectrophotometer (Thermo Scientific, ND-1000). SuperScript IV first-strand cDNA synthesis kit (Fisher, 18091050) was used for reverse transcription of RNA templates. Gene-specific primers targeted to *Homo sapiens* (H.s.) *TPI*, *HIF1A*, *HIF1-AS*, and the housekeeping gene β-actin (*ACTB*) were created by Integrated DNA Technologies (IDT) (Table S4). Reverse transcriptase (RT) reaction mix including 5× SuperScript IV Buffer, 100 mM DTT, ribonuclease inhibitor and SuperScript IV RT was added to RNA-primer mixes for each target. No RT samples were prepared by combining RNA-primer mixes with RT reaction mix including all reagents except RT. Combined reaction mixtures were heat blocked at 50°C for 10 min, then inactivated at 80°C for 10 min.

To prepare samples for PCR amplification, PowerTrack SYBR Green Master Mix (Fisher, A46012) kit was used following manufacturer's instructions. Samples, ‘no RT’ controls, and vehicle controls were plated on a 96-well plate in quadruplicate and sealed with a micro-seal sheet before being placed into a CFX Opus 96 real-time PCR system (Bio-Rad, 17005940). Data were normalized to an internal control (*ACTB*) and our 1% DMSO control, and these values were used to calculate relative gene expression.

### Data analyses

Statistical tests were conducted using GraphPad Prism v10.6.1. Western blots and cellular activity data were analyzed using a two-tailed Mann–Whitney test to determine significance. Multiple cell line western blots were analyzed using a Kruskal–Wallis non-parametric statistical test for determining significance. For all data, the error calculated and depicted is standard deviation. All western blot data includes eight independent repeats. TPI activity experiments with vadadustat include three independent experiments, each containing four technical replicates. Adaptaquin TPI activity experiments included one experiment with eight technical replicates (*n*=8).

## Supplementary Material



10.1242/joces.264664_sup1 Supplementary information

Table S1. Raw data and analyses
