# Supplementary material for: Biophysical and biochemical studies support PHD inhibitor development as a TPI deficiency therapy
Source: J Cell Sci. 2026 May 13;139(9):jcs264664. doi: 10.1242/jcs.264664 (PMC13245896; doi:10.1242/jcs.264664)
Supplement: Supplementary information [file joces-139-264664-s1.pdf]

Figure 4B:

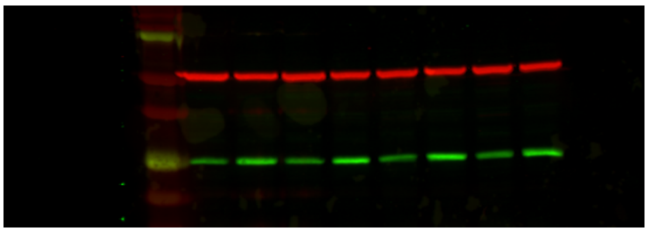

Figure 4D:

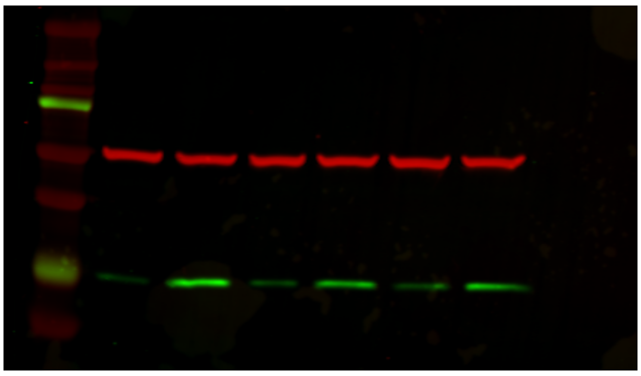

Figure 4F:

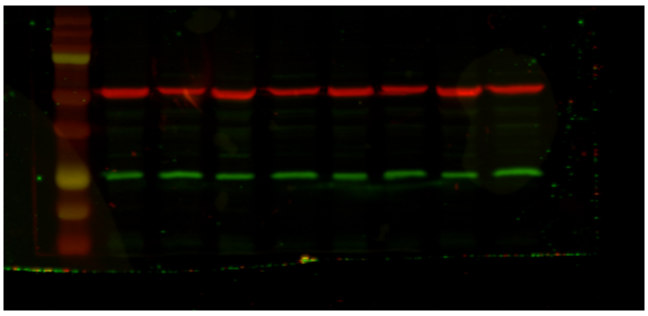

Figure 5:

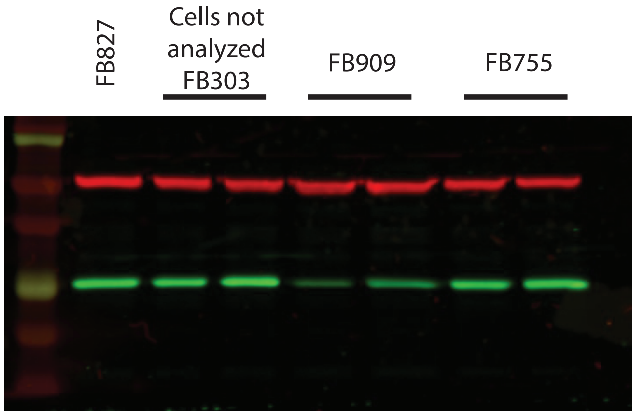

Fig. S1. Blot Transparency

Table S1. Raw data and analyses

Available for download at
https://journals.biologists.com/jcs/article-lookup/doi/10.1242/jcs.264664#supplementary-data

Table S2. Increased expression of several metabolic enzymes and known HIF target genes [7].

Gene	FB303 log2 fold	Adj. p-value	FB909 log2 fold	Adj. p-value
MIR210HG	4.52	<0.01	4.81	<0.01
HIF1A-AS2	4.55	<0.01	2.95	<0.01
VEGFA	3.47	<0.01	3.16	<0.01
ANGPTL4	3.76	<0.01	3.02	<0.01
DDIT4	3.11	<0.01	3.29	<0.01
ENO2	2.93	<0.01	2.46	<0.01
PFKFB4	3.04	<0.01	2.25	<0.01
ANKRD37	2.66	<0.01	2.61	<0.01
HK2	2.58	<0.01	2.55	<0.01
ALDOC	2.59	<0.01	2.32	<0.01
P4HA1	2.07	<0.01	2.18	<0.01
PGK1	2.24	<0.01	1.86	<0.01
GDF15	1.66	<0.01	1.85	<0.01
FAM162A	1.97	<0.01	1.51	<0.01
PDK1	1.62	<0.01	1.86	<0.01
MMP2	1.44	<0.01	2.03	<0.01
LDHA	1.56	<0.01	1.37	<0.01
HMOX1	1.63	<0.01	1.07	<0.01
GAPDH	1.59	<0.01	1.11	<0.01
P4HA2	1.40	<0.01	1.17	<0.01
PFKP	1.35	<0.01	1.08	<0.01
EGLN1	1.32	<0.01	1.14	<0.01
ALDOA	1.19	<0.01	1.09	<0.01

Journal of Cell Science • Supplementary information

Table S3. Disease-associated loci for which HIF activation is predicted to be beneficial.

Gene	FB303 log2 fold	FB909 log2 fold	Adj. p-value	Disease(s)	Inheritance	Refs.
**LEP**	6.19	4.91	<0.01	Congenital Leptin Deficiency	Autosomal Recessive	[1]
**EGR2**	3.86	2.38	<0.01	Charcot-Marie-Tooth neuropathy type 1D, Dejerine-Sottas neuropathy, and congenital hypomyelinating neuropathy.	All Mendelian types of inheritance observed	[2]
**TFR2**	3.62	*	<0.01	TFR2-Related Hemochromatosis	Autosomal Recessive	[3]
**NDRG1**	3.22	2.67	<0.01	Charcot-Marie-Tooth Neuropathy type 4D	Autosomal Recessive	[4]
**VLDLR**	2.77	2.42	<0.01	VLDLR-associated cerebellar hypoplasia, Dysequilibrium Synd.	Autosomal Recessive	[5]
**PLOD2**	2.3	2.18	<0.01	Bruck syndrome type II	Autosomal Recessive	[6]
**PGK1**	2.24	1.86	<0.01	Phosphoglycerate kinase deficiency	X-Linked Recessive	[7]
**SLC2A1**	2.24	2.26	<0.01	GLUT1 Deficiency Syndrome	Autosomal Recessive	[8]
**RORA**	2.03	1.78	<0.01	Intellectual Developmental Disorder with or without Epilepsy or Cerebellar Ataxia (IDDECA)	Auto. Dominant Happloinsufficiency	[9]
**JAM2**	1.7	1.16	<0.01	Primary familial brain calcification	Autosomal Recessive	[10]
**PAPPA2**	1.69	2.68	<0.01	Postnatal short stature, Dauber-Argente type	Autosomal Recessive	[11]
**MAN2B2**	*	1.64	<0.02	Congenital Disorder of Glycosylation type 1EE with or without immunodeficiency	Autosomal Recessive	[12]
**HMOX1**	1.63	1.07	<0.01	HMOX1 Deficiency	Autosomal Recessive	[13]
**LDHA**	1.56	1.37	<0.01	Glycogen Storage Disease XI	Autosomal Recessive	[14]
**GAA**	*	1.55	<0.01	Pompe Disease	Autosomal Recessive	[15]
**NOTCH3**	1.5	1.28	<0.01	Early Onset Arteriopathy	Autosomal Recessive	[16]
**TPI1**	1.48	*	<0.01	TPI Deficiency	Autosomal Recessive	[17]
**VWA1**	2.58	1.5	<0.01	Neuronopathy, distal hereditary motor, autosomal recessive 7	Autosomal Recessive	[18]
**MMP2**	1.44	2.03	<0.01	Winchester, Nodulosis-Arthropathy-Osteolysis syndromes	Autosomal Recessive	[19]
**ITGB3**	1.42	1.14	<0.01	Glanzmann thrombasthenia	Autosomal Recessive	[20]
**SLC6A9**	1.35	1.39	<0.01	Non-ketotic hyperglyinemia	Autosomal Recessive	[21]
**CTSF**	1.32	1.51	<0.01	Ceroid lipofuscinosis, neuronal, 13 (Kufs type)	Autosomal Recessive	[22]
**SERPING1**	1.27	1.88	<0.01	Hereditary angioedema (HAE)	Auto. Dom. Happloinsuff.	[23]
**DYSF**	1.26	1.14	<0.01	Miyoshi Myopathy, Limb Girdle Musc. Dystrophy 2B (LGMD2B)	Autosomal Recessive	[24]
**ATP6AP2**	*	1.25	<0.01	ATP6AP2-congenital disorder of glycosylation	X-Linked Recessive	[25]
**CRLF1**	1.14	1.34	<0.01	Crisponi Syndrome, Cold-Induced Sweating Syndrome Type 1	Autosomal Recessive	[26]
**PLOD1**	1.07	1.45	<0.01	PLOD1-Related Kyphoscoliotic Ehlers-Danlos Syndrome	Autosomal Recessive	[27]
**HEXA**	1.07	1.64	<0.01	Tay-Sachs Disease	Autosomal Recessive	[28]

* These loci are also upregulated, however, in the untreated samples the levels were too low for accurate quantification. Thus, they are increased by vadadustat treatment but the fold increase cannot be calculated.

Journal of Cell Science • Supplementary information

Table S4. Primers used in this study.

Primer (H.s.)	Sequence
TPI forward	5'-CTGGCATGATCAAAGACTGCGG-3'
TPI reverse	5'-CTCTGAGCCACCGCATCAGAGACGTTGG-3'
TPI RT	5'-GGCTTCTGGGCTGCTTAGTCCCTGG-3'
HIF-1a forward	5'-CCAAAAGAGGTGGATATGTCTGGG-3'
HIF-1a reverse	5'-GGCTTTGGCGTTTCAGCGGTGGG-3'
HIF-1a RT	5'-CTAGGTGTCTGATCCTGAATCTGG-3'
HIF1-AS2 forward	5'-GGACTCAACATACATTAAGGTGATGG-3'
HIF1-AS2 reverse	5'-CCTAAATGTTCTGCCTACCCTGTTGG-3'
HIF1-AS2 RT	5'-CAATACCCTATGTAGTTGTGGAAG-3'
ACTB forward	5'-CACAGAGCCTCGCCTTTGCCG-3'
ACTB reverse	5'-CAGCCAGGTCCAGACGCAGGATG-3'
ACTB RT	5'-GATGACCTGGCCGTCAGGCAG-3'

Table S5. Data Collection and Refinement

Statistics	Human TPI R5G
PDB ID:	
**Data collection**	
Space group	P 2(1) 2(1) 2(1)
Cell dimensions	
a, *b*, *c* (Å)	65.213 73.008 93.030
Unique Reflections	185,500
Resolution (Å)	57.434 – 1.065 (1.103-1.065)[a]
CC(1/2)	0.997 (0.083)
I / σI	9.5 (0.8)
Completeness (%)	93.7 (61.3)
Redundancy	6.0
**Refinement**	
Resolution (Å)	20.0 – 1.15 (1.191-1.150)
R_{work}[b] / R_{free}[c] (%)	12.37/13.75 (18.80/18.82)
Number of non-hydrogen atoms	
Protein	3,780
Solvent and Ligands	604
B-factors (Å^2)	
Protein	15.53
Solvent	25.53
R.m.s. deviations	
Bond lengths (Å)	0.007
Bond angles (°)	0.944
Ramachandrian	
Outliers (%)	0.0
Clashscore	0.13

[a]Values in parentheses are for highest-resolution shell.

[b]$R_{work} = \Sigma_{hkl}||F_{obs}(hkl)|| - F_{calc}(hkl)||/\Sigma_{hkl}|F_{obs}(hkl)|$.

[c]R_{free} represents the cross-validation R factor for 1.1% (~1,690) of the reflections against which the model was not refined.

References

[1] H.Yupanqui-Lozno, R.A. Bastarrachea, M.E. Yupanqui-Velazco, M. Alvarez-Jaramillo, E. Medina-Mendez, A.P. Giraldo-Pena, A. Arias-Serrano, C. Torres-Forero, A.M. Garcia-Ordonez, C.A. Mastronardi, C.M. Restrepo, E. Rodriguez-Ayala, E.J. Nava-Gonzalez, M. Arcos-Burgos, J.W. Kent, Jr., S.A. Cole, J. Licinio, L.G. Celis-Regalado, Congenital Leptin Deficiency and Leptin Gene Missense Mutation Found in Two Colombian Sisters with Severe Obesity, Genes (Basel) 10(5) (2019).

[2] D. Safka Brozkova, S. Nevsimalova, R. Mazanec, B. Rautenstrauss, P. Seeman, Charcot-Marie-Tooth neuropathy due to a novel EGR2 gene mutation with mild phenotype--usefulness of human mapping chip linkage analysis in a Czech family, Neuromuscul Disord 22(8) (2012) 742-6.

[3] M. Marco De Gobbi, PhD and Antonella Roetto, PhD., TFR2-Related Hemochromatosis, GeneReviews® [Internet].Initial Posting: August 29, 2005; Last Update: December 7, 2023.

[4] F.S. Skedsmo, A. Espenes, M.A. Tranulis, K. Matiasek, G. Gunnes, I. Bjerkas, L. Moe, S.S. Roed, M. Berendt, M. Fredholm, C. Rohdin, G.D. Shelton, P. Bruheim, M.H. Stafsnes, Z. Bartosova, L.C. Hermansen, O. Stigen, K.H. Jaderlund, Impaired NDRG1 functions in Schwann cells cause demyelinating neuropathy in a dog model of Charcot-Marie-Tooth type 4D, Neuromuscul Disord 31(1) (2021) 56-68.

[5] K.M. Boycott, C. Bonnemann, J. Herz, S. Neuert, C. Beaulieu, J.N. Scott, A. Venkatasubramanian, J.S. Parboosingh, Mutations in VLDLR as a cause for autosomal recessive cerebellar ataxia with mental retardation (dysequilibrium syndrome), J Child Neurol 24(10) (2009) 1310-5.

[6] S. Mumm, G.S. Gottesman, D. Wenkert, P.M. Campeau, A. Nenninger, M. Huskey, V.N. Bijanki, D.J. Veis, A.M. Barnes, J.C. Marini, M. Stolina, F. Zhang, W.H. McAlister, M.P. Whyte, Bruck syndrome 2 variant lacking congenital contractures and involving a novel compound heterozygous PLOD2 mutation, Bone 130 (2020) 115047.

[7] https://medlineplus.gov/genetics/condition/phosphoglycerate-kinase-deficiency/#inheritance.

[8] Glut1 Df. https://medlineplus.gov/genetics/condition/glut1-deficiency-syndrome/.

[9] OMIM: INTELLECTUAL DEVELOPMENTAL DISORDER WITH OR WITHOUT EPILEPSY OR CEREBELLAR ATAXIA; IDDECA. https://omim.org/entry/618060.

[10] Z. Cen, Y. Chen, S. Chen, H. Wang, D. Yang, H. Zhang, H. Wu, L. Wang, S. Tang, J. Ye, J. Shen, H. Wang, F. Fu, X. Chen, F. Xie, P. Liu, X. Xu, J. Cao, P. Cai, Q. Pan, J. Li, W. Yang, P.F. Shan, Y. Li, J.Y. Liu, B. Zhang, W. Luo, Biallelic loss-of-function mutations in JAM2 cause primary familial brain calcification, Brain 143(2) (2020) 491-502.

[11] OMIM: SHORT STATURE, DAUBER-ARGENTE TYPE; SSDA. https://omim.org/entry/619489.

[12] OMIM: MANNOSIDASE, ALPHA, CLASS 2B, MEMBER 2; MAN2B2. https://omim.org/entry/618899.

[13] G. Kovtunovych, M.C. Ghosh, W. Ollivierre, R.P. Weitzel, M.A. Eckhaus, J.F. Tisdale, A. Yachie, T.A. Rouault, Wild-type macrophages reverse disease in heme oxygenase 1-deficient mice, Blood 124(9) (2014) 1522-30.

[14] OMIM: LACTATE DEHYDROGENASE A; LDHA. https://www.omim.org/entry/150000.

[15] S. Taverna, G. Cammarata, P. Colomba, S. Sciarrino, C. Zizzo, D. Francofonte, M. Zora, S. Scalia, C. Brando, A.L. Curto, E.M. Marsana, R. Olivieri, S. Vitale, G. Duro, Pompe disease: pathogenesis, molecular genetics and diagnosis, Aging (Albany NY) 12(15) (2020) 15856-15874.

[16] I. Mizuta, Y. Nakao-Azuma, H. Yoshida, M. Yamaguchi, T. Mizuno, Progress to Clarify How NOTCH3 Mutations Lead to CADASIL, a Hereditary Cerebral Small Vessel Disease, Biomolecules 14(1) (2024).

[17] A. Williams, M. Weisz-Hubshman, V. Rossi, E. Bland, E. Mizerik, X. Luo, P.R. Hillman, K. Shields, F. Scaglia, TPI deficiency: A case report and review of the literature, Mol Genet Metab 146(1-2) (2025) 109227.

[18] OMIM: VON WILLEBRAND FACTOR A DOMAIN-CONTAINING PROTEIN 1; VWA1. https://omim.org/entry/611901.

[19] D. Wenkert, S. Mumm, S.M. Wiegand, W.H. McAlister, M.P. Whyte, Absence of MMP2 mutation in idiopathic multicentric osteolysis with nephropathy, Clin Orthop Relat Res 462 (2007) 80-6.

[20] Z. Wang, Y. Xu, Y. Sun, S. Wang, M. Dong, Novel homozygous silent mutation of ITGB3 gene caused Glanzmann thrombasthenia, Front Pediatr 10 (2022) 1062900.

[21] M. Alfadhel, M. Nashabat, H.A. Qahtani, A. Alfares, F.A. Mutairi, H.A. Shaalan, G.V. Douglas, K. Wierenga, J. Juusola, M.T. Alrifai, S.T. Arold, F. Alkuraya, Q.A. Ali, Mutation in SLC6A9 encoding a glycine transporter causes a novel form of non-ketotic hyperglycinemia in humans, Hum Genet 135(11) (2016) 1263-1268.

Journal of Cell Science • Supplementary information

[22] OMIM: CATHEPSIN F; CTSF. https://omim.org/entry/603539.
[23] A.K.H. Alfonso Rodriguez Espada, Sean J. Jurgens, Sharjeel A Chaudhry, Justine Ryu, Seung Hoan Choi, Harish Eswaran, Simone Sanna-Cherchi, Patrick Ellinor, Steven Grover, Pavan K Bendapudi, Rare Germline Loss-of-Function Variants in C1-Inhibitor (SERPING1) Are Associated with Venous Thromboembolism in Population-Scale Sequencing Datasets, Blood 144 (2024) 1196.
[24] C. Bouchard, J.P. Tremblay, Portrait of Dysferlinopathy: Diagnosis and Development of Therapy, J Clin Med 12(18) (2023).
[25] OMIM: CONGENITAL DISORDER OF GLYCOSYLATION, TYPE IIr; CDG2R. https://omim.org/entry/301045.
[26] L. Crisponi, G. Crisponi, A. Meloni, M.R. Toliat, G. Nurnberg, G. Usala, M. Uda, M. Masala, W. Hohne, C. Becker, M. Marongiu, F. Chiappe, R. Kleta, A. Rauch, B. Wollnik, F. Strasser, T. Reese, C. Jakobs, G. Kurlemann, A. Cao, P. Nurnberg, F. Rutsch, Crisponi syndrome is caused by mutations in the CRLF1 gene and is allelic to cold-induced sweating syndrome type 1, Am J Hum Genet 80(5) (2007) 971-81.
[27] MEDLINE: Plod1. https://medlineplus.gov/genetics/gene/plod1/#conditions.
[28] OMIM: HEXOSAMINIDASE A; HEXA. https://www.omim.org/entry/606869.

Journal of Cell Science • Supplementary information
