# Supplementary material for: Biophysical and biochemical studies support PHD inhibitor development as a TPI deficiency therapy
Source: J Cell Sci. 2026 May 13;139(9):jcs264664. doi: 10.1242/jcs.264664 (PMC13245896; doi:10.1242/jcs.264664)
Supplement: Supplementary file 1 [file joces-139-264664_review_history.pdf]

Journal of Cell Science

Biophysical and biochemical studies support PHD inhibitor development as a TPI deficiency therapy

Presley Roberts, Joseph R. Figura, Kaitlin McClure, Grace Coleman, Cindy Cui, Riley Sawka, Laura L. Vollmer, Andreas Vogt, Ariana J. Jou, Andrew P. VanDemark and Michael J. Palladino
DOI: 10.1242/jcs.264664

Editor: Simon Cook

Review timeline
Original submission: 15 December 2025
Editorial decision: 5 February 2026
First revision received: 12 February 2026
Accepted: 29 March 2026

Original submission

First decision letter

MS ID#: jcs.264664

MS TITLE: Biophysical and Biochemical Studies Support PHD Inhibitor Development as a TPI Deficiency Therapy

AUTHORS: Presley Roberts; Joseph R Figura; Kaitlin McClure; Grace Coleman; Cindy Cui; Riley Sawka; Laura L Vollmer; Andreas Vogt; Ariana J Jou; Andrew P VanDemark; Michael J Palladino

ARTICLE TYPE: Research Article

Dear Dr Palladino,

We have now reached a decision on the above manuscript.

As you will see, the reviewers raise a number of substantial criticisms that prevent me from accepting the paper at this stage. Looking at their comments I believe a revised version might prove acceptable, if you can address their concerns. If you think that you can deal satisfactorily with the criticisms on revision, I would be pleased to see a revised manuscript. We would then return it to the reviewers.

Reviewer 1

Advance summary and potential significance to field

Roberts et al. present a crystal structure of a disease-causing mutant of triosephosphate isomerase (TPI) and explore the potential for prolyl hydroxylase domain (PHD) inhibitors as a therapeutic strategy for TPI deficiency. Building on their previous work, the authors use a series of in vitro assays and patient-derived cell models to show that PHD inhibition increases TPI protein abundance and is associated with increased TPI activity. They also report induction of a putative negative regulator of HIF signalling following PHD inhibitor treatment. While several experiments are well controlled and the structural component is of interest, the overall manuscript currently lacks a

© 2026. Published by The Company of Biologists under the terms of the Creative Commons Attribution License (https://creativecommons.org/licenses/by/4.0/).

cohesive narrative, and a number of findings appear preliminary or insufficiently developed to support the conclusions as written.

Comments for the author

Major comments
Overall narrative, novelty and translational relevance
My primary concern is the lack of a clear and coherent central narrative connecting the structural findings to the proposed therapeutic implications. The authors show that PHD inhibitors increase TPI protein levels and enzymatic activity, which is presumed to occur through HIF-dependent transcriptional mechanisms. However, the manuscript additionally introduces HIF-1AS2 and a potential negative feedback model that is not integrated into the main story and is not explored in sufficient depth. As a consequence, the conclusions remain largely limited to HIF-mediated upregulation of TPI, conceptually consistent with existing knowledge, alongside the suggestion that PHD inhibition may be beneficial in TPI deficiency without providing compelling functional evidence. The manuscript would be substantially strengthened by demonstrating that PHD inhibitors improve a biologically or clinically meaningful phenotype in a disease-relevant model (e.g., patient-derived cells). In its current form, the rationale for including HIF-1AS2 is unclear, as the data presented are insufficient to establish a mechanistic contribution or robust conclusion.

Figure 3: Use of nuclear size as a toxicity readout
In Figure 3, the authors use an IHC-based approach to quantify TPI levels alongside changes in nuclear size across doses of PHD inhibitor. Nuclear size can be influenced by multiple factors beyond toxicity, including cell cycle distribution and stress responses. The authors should support any claims relating to cellular health or toxicity with established viability and apoptosis assays (e.g., Annexin V/PI staining, caspase activity assays, or standard metabolic viability assays).

Figure 4: Evidence of pathway engagement, dose-dependence and kinetics
In Figure 4, TPI levels are assessed by Western blot following treatment with a single inhibitor concentration, but there is no evidence provided that the compound engages the intended pathway (e.g., HIF stabilisation). Demonstrating HIF-1Î± stabilisation would confirm on-target activity. Moreover, a time-course analysis would clarify the kinetics of HIF induction and whether changes in TPI follow expected temporal dynamics. A dose-response Western blot would further strengthen the conclusion by demonstrating concentration-dependent effects on TPI abundance.

Figure 5: Interpretation of enzymatic activity data
The activity assay in Figure 5 lacks sufficient methodological detail to fully assess the robustness of the measurements. In addition, given that the authors already show increased TPI protein abundance (Figure 4), the conclusion that "TPI activity is increased" should be interpreted cautiously. The observed increase in total activity may primarily reflect increased protein abundance rather than altered specific activity. The authors should clarify whether activity has been normalised to TPI protein levels and/or report specific activity. Framing this correctly is particularly important in a disease context where increased abundance may still be beneficial, but the mechanistic interpretation differs.

Figure 6: Functional benefit in patient-derived cells
In Figure 6, the authors demonstrate increased TPI levels in a patient-derived cell line following treatment. However, the translational relevance would be significantly strengthened by showing that this molecular change confers a functional advantage. The authors should consider adding disease-relevant functional assays (e.g., cellular performance metrics, metabolic outputs, stress tolerance, or other relevant endpoints) to demonstrate benefit rather than protein induction alone.

Figure 7B: Missing baseline control and lack of protein validation
In Figure 7B, the untreated (baseline) time point should be included to enable proper interpretation of RNA changes over time. More broadly, transcript changes do not necessarily correlate with protein abundance, and this is especially true for HIF-1Î±, which is predominantly regulated post-translationally via VHL-dependent degradation. The authors should therefore include protein measurements (e.g., Western blots) of TPI and HIF-1Î± over the same time course to support conclusions regarding pathway activation and downstream effects.

© 2026. Published by The Company of Biologists under the terms of the Creative Commons Attribution License (https://creativecommons.org/licenses/by/4.0/).

Minor comments
Abstract clarity regarding "lead compounds"
The abstract states that a high-throughput screen identified lead compounds, but it is not clear what these compounds are "leads" for (e.g., PHD inhibitors, modulators of TPI stability/activity, or another target class). This should be clarified.

Justification and framing of HIF-1AS2
The manuscript states that RNA-sequencing identified HIF1-AS2 as an induced target, but it does not explain why this is important or how it advances the study's conclusions. As presented, this element detracts from the main message unless mechanistically developed or repositioned.

Typographical correction
Line 62: "uMM" should be corrected to "μM".

Reviewer 2

Advance summary and potential significance to field

This is a strong manuscript that presents genuine novelty and clear mechanistic insight. The findings may lead to relevant translational implications. The data reported herein presented suggests that repurposing of prolyl hydroxylase domain inhibitors (PHDIs) may be a potential therapy for TPI Df. The crystal structure of TPIR5G demonstrates a structurally normal protein except for the very n-terminus, implying that is sufficient to lead to a reduction in stability conducive to strikingly reduced TPI steady state levels. In addition, the authors demonstrate that chronic PHDI treatment leads to activation of a HIF-1a/HIF1-AS2 feedback loop, which has implications for dosing and treatment. This approach may also have implications for several chronic diseases where sustained HIF activation is needed. However, the authors need to revise the manuscript accordingly before it would be suitable for publication.

Comments for the author

Major comments: The main question is that it seems all cellular experiments use a concentration of 100 μM PHDIs, which is orders of magnitude above clinically achievable plasma levels for drugs like vadadustat, roxadustat, and other PHDI. Although this is acceptable as a proof-of-concept, this needs to be acknowledged in the discussion section of the manuscript. A paragraph should be added to the discussion explicitly addressing the known clinical Cmax values and the disparity needs to be acknowledged. A lower-dose chronic exposure to this type of medications or combination therapy may be more doable in clinical studies. It should be emphasized that these results emphasize mechanistic validation, without direct clinical translation at this stage.

HIF1-AS2 is presented as a negative feedback regulator of HIF-1Î±. This is supported by the literature, but could the downregulation of HIF-1Î± be multifactorial? This possibility should be entertained.

Claims about quality control recognition and moonlighting functions for this protein are speculative and therefore they should be labeled as hypotheses.

In this manuscript, the authors should focus on TPI Df and not trying to extend conclusions to additional diseases. This simply detracts from the focus of the paper. It is interesting and it could be briefly mentioned in the discussion but it looks overly expansive. A sentence should be added stating that these are hypothesis-generating observations and functional validation is required. Table 1 is very distracting and should be changed into a Supplementary Table as the focus on the manuscript should be TPI Df.

Consider moving Table 1 emphasis

Minor comments

Was there any statistical cutoff criteria explicitly used for RNA seq?

© 2026. Published by The Company of Biologists under the terms of the Creative Commons Attribution License (https://creativecommons.org/licenses/by/4.0/).

Please use HIF-1Î± consistently and avoid switching between HIF-1a / HIF1a. Please be consistent and use either PHDI or PHD inhibitor consistently per section.

First revision

Author response to reviewers' comments

We thank both reviewers for taking the time to read our manuscript and provide constructive comments and suggestions. We have provided a point-by-point response to all reviewer comments below in blue.

Reviewer 1: SUMMARY OF THE ADVANCE MADE IN THIS PAPER AND ITS POTENTIAL SIGNIFICANCE TO THE FIELD

Roberts et al. present a crystal structure of a disease-causing mutant of triosephosphate isomerase (TPI) and explore the potential for prolyl hydroxylase domain (PHD) inhibitors as a therapeutic strategy for TPI deficiency. Building on their previous work, the authors use a series of in vitro assays and patient-derived cell models to show that PHD inhibition increases TPI protein abundance and is associated with increased TPI activity. They also report induction of a putative negative regulator of HIF signalling following PHD inhibitor treatment. While several experiments are well controlled and the structural component is of interest, the overall manuscript currently lacks a cohesive narrative, and a number of findings appear preliminary or insufficiently developed to support the conclusions as written.

We have improved the cohesive narrative making it more clear to the reader that the structure being normal, with the exception of the very N-terminus, and bearing activity allows us to test new therapies that increase mutant protein levels (as the mutant protein is functional) and that the negative HIF feedback loop we describe will need to be avoided for this therapeutic approach to be viable.

SUGGESTIONS TO AUTHORS

Major comments
Overall narrative, novelty and translational relevance
My primary concern is the lack of a clear and coherent central narrative connecting the structural findings to the proposed therapeutic implications. The authors show that PHD inhibitors increase TPI protein levels and enzymatic activity, which is presumed to occur through HIF-dependent transcriptional mechanisms. However, the manuscript additionally introduces HIF-1AS2 and a potential negative feedback model that is not integrated into the main story and is not explored in sufficient depth. As a consequence, the conclusions remain largely limited to HIF-mediated upregulation of TPI, conceptually consistent with existing knowledge, alongside the suggestion that PHD inhibition may be beneficial in TPI deficiency without providing compelling functional evidence. The manuscript would be substantially strengthened by demonstrating that PHD inhibitors improve a biologically or clinically meaningful phenotype in a disease-relevant model (e.g., patient-derived cells). In its current form, the rationale for including HIF-1AS2 is unclear, as the data presented are insufficient to establish a mechanistic contribution or robust conclusion.

While there is published data about the existence of a HIF1-AS2 feedback loop, which we cite, the finding that this can be activated by a single PHDI treatment and its relevance to treating chronic diseases like TI Df are new and support the importance of including these data in the manuscript. We have modified our description of these results to more carefully relay their relevance and to accurately describe them as initial findings deserving of additional research.

The comment that “manuscript would be substantially strengthened by demonstrating that PHD inhibitors improve a biologically or clinically meaningful phenotype in a disease-relevant model

© 2026. Published by The Company of Biologists under the terms of the Creative Commons Attribution License (https://creativecommons.org/licenses/by/4.0/).

(e.g., patient-derived cells)" is concerning. Almost every experiment reported was performed in TPI Df patient derived cells and we show that mutant TPI protein itself, as well as its activity, are increased. We agree the fact that almost all of this research was performed in patient cells is especially important and have edited the manuscript to make this even more clear.

Figure 3: Use of nuclear size as a toxicity readout
In Figure 3, the authors use an IHC-based approach to quantify TPI levels alongside changes in nuclear size across doses of PHD inhibitor. Nuclear size can be influenced by multiple factors beyond toxicity, including cell cycle distribution and stress responses. The authors should support any claims relating to cellular health or toxicity with established viability and apoptosis assays (e.g., Annexin V/PI staining, caspase activity assays, or standard metabolic viability assays).

While changes in nuclear size are an important indicator of toxicity and should be evaluated as we have done, we agree other assays of toxicity could be investigated. While we support the idea that rigorous tests for toxicity can be valuable, we must carefully weigh the scientific merit of such experiments against the value of a precious and finite resource such as patient-derived fibroblasts that were donated by very sick children. The main rationale for using an image-based toxicity readout was to ensure data are rigorous and did not confound measurements of TPI levels due to changes in cell and nuclear morphology, not to carefully validate the nature or mechanism of potential cytotoxicity. Furthermore, much of the results we report focus on vadadustat, which is approved for use in the US and EU and Roxadustat, which is approved in multiple countries in Asia. As such, these drugs have undergone extensive toxicity and safety tests and together have been used safely in millions of humans for the treatment of CKD. We believe our reporting that these drugs are not causing obvious toxicity in TPI Df cells is merited but that further tests of toxicity with these drugs is not merited.

Figure 4: Evidence of pathway engagement, dose-dependence and kinetics
In Figure 4, TPI levels are assessed by Western blot following treatment with a single inhibitor concentration, but there is no evidence provided that the compound engages the intended pathway (e.g., HIF stabilisation). Demonstrating HIF-1α stabilisation would confirm on-target activity. Moreover, a time-course analysis would clarify the kinetics of HIF induction and whether changes in TPI follow expected temporal dynamics. A dose-response Western blot would further strengthen the conclusion by demonstrating concentration-dependent effects on TPI abundance.

While demonstrating HIF stabilization is something that could be done, the MOA of PHDI's is very well established and we test a panel of these drugs that all function similarly, making such experiments less critical. We use a state-of-the-art image based, validated assay to perform a 10-point dose response, which is the standard in the field and uses only a small fraction of patient cells as compared to a western blot (WB). Importantly, we do validate our image based system using WBs as an orthogonal assay, using a dosage that provides us with an independent measure of the maximal change observed in TPI. Not only would dose-response WBs require more cells that we have, but they would also not be an ethical use of patient cells as they would not even incrementally improve the results being reported. Time-courses for HIF-1alpha and TPI are shown by qRT-PCR in Figure 7, with fast (1h) and transient activation of *HIF-1a* , leading to later expression of *TPI*, as expected from the literature.

Figure 5: Interpretation of enzymatic activity data
The activity assay in Figure 5 lacks sufficient methodological detail to fully assess the robustness of the measurements. In addition, given that the authors already show increased TPI protein abundance (Figure 4), the conclusion that "TPI activity is increased" should be interpreted cautiously. The observed increase in total activity may primarily reflect increased protein abundance rather than altered specific activity. The authors should clarify whether activity has been normalised to TPI protein levels and/or report specific activity. Framing this correctly is particularly important in a disease context where increased abundance may still be beneficial, but the mechanistic interpretation differs.

We agree with that it is important to distinguish between increases in activity and specific activity. The reviewer is correct that the observed increase in TPI activity is most likely a result of increased expression levels, as the purified protein is structurally normal and exhibits wild-type activity *in vitro*. To clarify this conclusion, we report the activity normalized to total cellular protein - not to

© 2026. Published by The Company of Biologists under the terms of the Creative Commons Attribution License (https://creativecommons.org/licenses/by/4.0/).

TPI protein - and have modified our description to ensure readers know we are not presenting a measurement of specific activity, but rather an activity measurement normalized to total protein concentration.

Figure 6: Functional benefit in patient-derived cells
In Figure 6, the authors demonstrate increased TPI levels in a patient-derived cell line following treatment. However, the translational relevance would be significantly strengthened by showing that this molecular change confers a functional advantage. The authors should consider adding disease-relevant functional assays (e.g., cellular performance metrics, metabolic outputs, stress tolerance, or other relevant endpoints) to demonstrate benefit rather than protein induction alone.

TPI Df is well established to result from insufficient TPI protein and activity, which we report. Any other assays would be less direct and less valuable, especially when performed in vitro. Tests in animal models are clearly beyond the scope of this manuscript.

Figure 7B: Missing baseline control and lack of protein validation
In Figure 7B, the untreated (baseline) time point should be included to enable proper interpretation of RNA changes over time. More broadly, transcript changes do not necessarily correlate with protein abundance, and this is especially true for HIF-1α, which is predominantly regulated post-translationally via VHL-dependent degradation. The authors should therefore include protein measurements (e.g., Western blots) of TPI and HIF-1α over the same time course to support conclusions regarding pathway activation and downstream effects.

While we understand an untreated measurement would help interpret the RNA changes overtime, this does not negate the value of the results reported and further studies cannot be justified in these patient cells. As was suggested by Reviewer 2, we have revised the manuscript to clarify that these are hypothesis generating experiments and require further validation.

Minor comments
Abstract clarity regarding "lead compounds"
The abstract states that a high-throughput screen identified lead compounds, but it is not clear what these compounds are "leads" for (e.g., PHD inhibitors, modulators of TPI stability/activity, or another target class). This should be clarified.

It was clarified. Thank you.

Justification and framing of HIF-1AS2
The manuscript states that RNA-sequencing identified HIF1-AS2 as an induced target, but it does not explain why this is important or how it advances the study's conclusions. As presented, this element detracts from the main message unless mechanistically developed or repositioned.

We improved our description and framing. Thank you.

Typographical correction
Line 62: "uMM" should be corrected to "µM".

This was corrected. Thank you.

Reviewer 2:
SUMMARY OF THE ADVANCE MADE IN THIS PAPER AND ITS POTENTIAL SIGNIFICANCE TO THE FIELD

This is a strong manuscript that presents genuine novelty and clear mechanistic insight. The findings may lead to relevant translational implications. The data reported herein presented suggests that repurposing of prolyl hydroxylase domain inhibitors (PHDIs) may be a potential therapy for TPI Df. The crystal structure of TPIR5G demonstrates a structurally normal protein except for the very n-terminus, implying that is sufficient to lead to a reduction in stability conducive to strikingly reduced TPI steady state levels. In addition, the authors demonstrate that chronic PHDI treatment leads to activation of a HIF-1a/HIF1-AS2 feedback loop, which has implications for dosing and treatment. This approach may also have implications for several chronic

© 2026. Published by The Company of Biologists under the terms of the Creative Commons Attribution License (https://creativecommons.org/licenses/by/4.0/).

diseases where sustained HIF activation is needed. However, the authors need to revise the manuscript accordingly before it would be suitable for publication.

We thank the reviewer for their constructive comments and appreciate the positive evaluation of novelty and significance as we believe we have uncovered a promising venue that could potentially lead to repurposed drugs.

SUGGESTIONS TO AUTHORS

Major comments: The main question is that it seems all cellular experiments use a concentration of 100 µM PHDIs, which is orders of magnitude above clinically achievable plasma levels for drugs like vadadustat, roxadustat, and other PHDI. Although this is acceptable as a proof-of-concept, this needs to be acknowledged in the discussion section of the manuscript. A paragraph should be added to the discussion explicitly addressing the known clinical Cmax values and the disparity needs to be acknowledged. A lower-dose chronic exposure to this type of medications or combination therapy may be more doable in clinical studies. It should be emphasized that these results emphasize mechanistic validation, without direct clinical translation at this stage.

Thank you for this comment. The main purpose of this work is indeed proof-of-concept to support a role for PHD inhibition in increasing levels of mutant TPI, and we now preface the corresponding section in the discussion accordingly. With regards to clinically achievable drug levels, only the western blots and complementary activity assays were done at 100 µM, and those served primarily to confirm increased expression of TPI by orthogonal and complementary assays. Our dose-response studies in patient cells by immunofluorescence showed that the compounds in question had EC50s in the low micromolar range, which is below what is achievable in studies in rats and in humans [1] [2]. We have updated the manuscript to include the published Cmax values of vadadustat following a single administration in rats and in patients that are significnatly above the EC50 we report that activate TPI expression and above the levels used in WB validation studies.

HIF1-AS2 is presented as a negative feedback regulator of HIF-1α. This is supported by the literature, but could the downregulation of HIF-1α be multifactorial? This possibility should be entertained.

We agree. Thank you . We have added a statement saying the downregulation of HIF-1a could be multifactorial.

Claims about quality control recognition and moonlighting functions for this protein are speculative and therefore they should be labeled as hypotheses.

We agree and have labeled the moonlighting functions and described the recognition of mutant protein by protein QC pathways as hypotheses, as suggested.

In this manuscript, the authors should focus on TPI Df and not trying to extend conclusions to additional diseases. This simply detracts from the focus of the paper. It is interesting and it could be briefly mentioned in the discussion but it looks overly expansive. A sentence should be added stating that these are hypothesis-generating observations and functional validation is required. Table 1 is very distracting and should be changed into a Supplementary Table as the focus on the manuscript should be TPI Df.

We have made the table of other diseases a Supplementary table as suggested. We have also added several sentences about the table to emphasize that these are hypothesis-generating observations that require deeper study and validation in other systems.

Minor comments

Was there any statistical cutoff criteria explicitly used for RNA seq?

We provide all the data so anyone can analyze it with whatever cutoff they want. We used corrected p-values of $p< 0.01$ as our cutoff. Adjusted p values are provided in Figure 7. The p-

© 2026. Published by The Company of Biologists under the terms of the Creative Commons Attribution License (https://creativecommons.org/licenses/by/4.0/).

values used as cutoffs in the Supplemental tables are described in the results (cutoff used was an adjusted p-value of p<0.01 in both cell lines).

Please use HIF-1α consistently and avoid switching between HIF-1a / HIF1a. Please be consistent and use either PHDI or PHD inhibitor consistently per section.

Corrected. Thank you.

References:

[1] A. Zuk, Z. Si, S. Loi, S. Bommegowda, D. Hoivik, S. Danthi, G. Molnar, V. Csizmadia, M. Rabinowitz, Preclinical Characterization of Vadadustat (AKB-6548), an Oral Small Molecule Hypoxia-Inducible Factor Prolyl-4-Hydroxylase Inhibitor, for the Potential Treatment of Renal Anemia, J Pharmacol Exp Ther 383(1) (2022) 11-24.

[2] FDA_Access_Data, 2024. https://www.accessdata.fda.gov/drugsatfda_docs/nda/2024/215192Orig1s000TOC.cfm.

Second decision letter

MS ID#: jcs.264664R1

MS Title: Biophysical and Biochemical Studies Support PHD Inhibitor Development as a TPI Deficiency Therapy

Authors: Presley Roberts; Joseph R Figura; Kaitlin McClure; Grace Coleman; Cindy Cui; Riley Sawka; Laura L Vollmer; Andreas Vogt; Ariana J Jou; Andrew P VanDemark; Michael J Palladino

Article Type: Research Article

Dear Dr Palladino,

I am happy to tell you that your manuscript has been accepted for publication in Journal of Cell Science, pending standard publication integrity checks.

© 2026. Published by The Company of Biologists under the terms of the Creative Commons Attribution License (https://creativecommons.org/licenses/by/4.0/).
